# Patient-Specific Spatio-Temporal False Data Injection Attack Detection for IoMT Using a Graph-GRU Digital Twin and Kalman Innovation Features

**DOI:** 10.3390/bioengineering13080920

**Published:** 2026-08-14

**Authors:** Eman H. Alkhammash, Fuad A. Ghaleb, Faisal Saeed, Sultan Noman Qasem

**Affiliations:** 1 Department of Computer Science, College of Computers and Information Technology, Taif University, Taif 21944, Saudi Arabia; eman.kms@tu.edu.sa; 2 Department of Computer Science, Birmingham City University, Birmingham B4 7XG, UK; fuad.ghaleb@bcu.ac.uk (F.A.G.); faisal.saeed@bcu.ac.uk (F.S.); 3 Computer Science Department, College of Computer and Information Sciences, Imam Mohammad Ibn Saud Islamic University (IMSIU), Riyadh 11432, Saudi Arabia

**Keywords:** internet of medical things, IoMT, false data injection attacks, FDIA, digital twin, graph convolutional network, gated recurrent unit, GCN–GRU, Kalman filter, anomaly detection, patient-specific monitoring, edge intelligence

## Abstract

The Internet of Medical Things (IoMT) is a promising technology for enabling smart and efficient healthcare systems through continuous physiological monitoring, early anomaly detection, and proactive patient management. However, IoMT sensors are vulnerable to False Data Injection Attacks (FDIAs), in which adversaries can manipulate sensor measurements to compromise diagnostic accuracy, mislead clinical decision-making, and threaten patient safety. Existing detection approaches often rely on population-level statistical models that may not fully capture individual physiological variations or residual-based thresholds designed for relatively simple attack scenarios, limiting their ability to exploit the spatio-temporal dependencies of multi-sensor physiological streams and detect stealthy or adversarial FDIAs. This paper proposes a patient-specific FDIA detection framework based on a Graph Convolutional Network–Gated Recurrent Unit (GCN–GRU) digital twin that learns an individual patient’s normal physiological behaviour from clean baseline telemetry. The trained digital twin is integrated into a Kalman filter as the state prediction model, and the resulting standardised innovation residuals are used as detection features. To characterise stealthy attack behaviours, four complementary window-based feature groups are extracted from the innovation sequence: innovation statistics, sensor correlation drift, temporal smoothness, and uncertainty mismatch. A CNN-1D classifier is then trained to learn discriminative temporal attack patterns from these features for accurate detection. A structured attack taxonomy comprising five stealthy and adversarial FDIA scenarios is developed, where attacks are injected as smooth gradual or abrupt coordinated modifications to sensor measurements while remaining within plausible physiological ranges. Experiments conducted on the WUSTL-EHMS-2020 benchmark dataset demonstrate that the proposed framework achieves an F1-score of 94.3%, outperforming Isolation Forest and PCA Reconstruction by 34 percentage points. Furthermore, the proposed framework reduces the false alarm rate to 3.6%, compared with 35.1% and 9.2% achieved by Isolation Forest and PCA Reconstruction, respectively. These results demonstrate the effectiveness of the proposed framework for reliable detection of stealthy FDIAs in IoMT-based healthcare systems.

## 1. Introduction

The rapid adoption of connected medical sensing devices within the Internet of Medical Things (IoMT) paradigm has revolutionised healthcare systems. Traditional health and medical systems have been proven ineffective and inefficient in timely responding to patient needs due to the increasing number of patients and limited resources [1,2,3]. In this context, IoMT provides an effective and efficient solution by enabling the continuous and real-time collection of physiological data, including blood pressure, heart rate, respiratory rate, blood oxygen saturation, skin temperature, and electrocardiographic signals, to support a broad range of healthcare applications including remote patient monitoring, early detection of anomalies in patient conditions, and personalised treatment adjustment [4]. The global IoMT market was valued at USD 44.2 billion in 2023 [5], while other market analyses project substantial growth of the broader IoT healthcare market, reaching USD 963.79 billion by 2030 [6]. In addition, the number of IoT-connected devices worldwide is expected to increase substantially in the coming years [7], while 59% of healthcare providers have reported implementing IoMT solutions in their organisations [7]. This rapid adoption of IoMT introduces significant security vulnerabilities due to the use of resource-constrained medical devices and potentially insecure wireless communication channels. These constraints can limit the implementation of advanced security mechanisms, increasing the risk of cyber-attacks that may compromise the integrity, reliability, and trustworthiness of physiological data and threaten patient safety. Various security solutions have been proposed for IoMT-based applications, including prevention, detection, and mitigation mechanisms [2,8,9]. However, false data injection remains underexplored in healthcare [9]. False Data Injection Attacks (FDIAs) have been recognised as a significant threat due to their ability to silently manipulate sensor measurements and misrepresent a patient’s actual physiological condition [10,11,12]. FDIAs can hide abnormal physiological changes, delaying the identification of emergencies such as cardiovascular events, respiratory deterioration, or other acute conditions [12].

FDIAs have been widely studied in traditional cyber-physical systems [13,14], including smart grids [15,16,17,18,19,20], industrial control systems [21,22], and wireless sensor networks [23,24]. However, there are limited studies conducted for IoMT and healthcare contexts. The need for domain-specific datasets for healthcare FDIAs has been highlighted in [9]. Various security mechanisms have been investigated, including prevention, mitigation, and detection approaches. However, prevention alone cannot eliminate the risk of compromised devices or manipulated data, making detection and mitigation essential. Several approaches have been proposed for detecting false data injections attacks in the literature since the first theoretical foundations of FDIAs were established by [25]. Existing detection approaches can be divided into model-based and data-driven methods. Model-based approaches, which use state estimation and are widely used in smart grids and ICS networks, have rarely suggested IoMT due to the absence of a generalised accurate state prediction model for human physiological data. Many studies [10,25] demonstrated that coordinated stealthy adversarial FDIAs with limited knowledge about the model can evade residual-based state estimation detectors and construct an attack-signature-based solution using temporal and spatial deviation of sensors data from physiological plausibility models. Data-driven approaches, including statistical methods and machine learning techniques, have been introduced to overcome these limitations by learning normal system behaviour from historical data and identifying abnormal patterns.

Several studies have demonstrated the potential of machine learning and anomaly detection methods for FDIA detection in IoMT systems [3,12,26,27,28]. However, these studies focus on network-level security threats, rely on predefined attack scenarios, or use population-level models that do not fully account for individual physiological differences. Limited research has addressed the modelling of patient-specific temporal and spatial relationships or the detection of stealthy and adversarial FDIAs that generate clinically plausible measurements while deviating from an individual’s normal physiological behaviour. These limitations indicate the need for patient-specific detection frameworks that can learn individual physiological dynamics, motivating the development of digital twin-based approaches for more robust FDIA detection in IoMT healthcare systems.

Detecting FDIAs in IoMT physiological streams remains challenging due to the patient-specific nature of physiological data, which are multivariate and temporally non-stationary [3,10]. Normal physiological patterns, variability, and inter-sensor relationships can differ significantly between individuals, limiting the effectiveness of population-based detection models [9]. Furthermore, stealthy, and adversarial FDIA strategies can exploit knowledge of the detection system and generate measurements that remain close to expected physiological behaviour, making them difficult to identify. Such attacks pose a direct risk to patient safety due to the ability of attackers to craft false data that remain within the normal operating range of physiological signals to evade conventional anomaly detection mechanisms, potentially leading to incorrect diagnoses or treatment decisions and compromising the integrity of physiological measurements [3]. Stealthy and adversarial FDIAs can remain within general clinical ranges while deviating from an individual patient’s normal physiological patterns, making them difficult to detect [29]. Methods developed for non-healthcare systems cannot be directly applied to IoMT because physiological behaviour is highly patient-specific and cannot be accurately represented by a single global model [9,10]. Effective detection therefore requires modelling the spatio-temporal characteristics of multivariate physiological data, as inconsistencies may only become evident through relationships across sensors and over time. Furthermore, existing detection approaches often rely on population-based statistical models or fixed residual thresholds, which may not fully account for inter-patient physiological variability. Wider thresholds may therefore be required to accommodate population variability, potentially reducing sensitivity to subtle deviations from an individual patient’s normal behaviour. As highlighted in [29], attacks may remain within general physiological norms while deviating from the patient’s personal signal characteristics. However, limited attention has been given to modelling patient-specific temporal and spatial relationships for detecting stealthy and adversarial FDIAs. These limitations motivate the use of patient-specific physiological modelling and digital-twin-based approaches for FDIA detection in IoMT healthcare systems.

To address this gap, this study proposes a patient-specific spatio-temporal FDIA detection framework for IoMT-based healthcare systems, integrating a Graph Convolutional Network–Gated Recurrent Unit (GCN–GRU) digital twin, Kalman innovation analysis, and a lightweight one-dimensional convolutional neural network (CNN-1D) classifier. The GCN–GRU digital twin is developed and employed to model each patient’s normal physiological behaviour by learning both spatial dependencies among physiological sensors and temporal dynamics of physiological signals. The digital twin operates as a shadow state to estimate the expected physiological condition, while Kalman filtering is used to generate standardised innovation features that quantify deviations between expected and observed measurements. A sliding-window feature extraction approach is applied to capture different stealthy and adversarial attack characteristics, including abnormal innovation patterns, sensor relationship changes, temporal inconsistencies, and uncertainty variations. An edge-based, lightweight CNN-1D classifier is then developed for efficient attack detection. The proposed framework is evaluated under various stealthy and adversarial FDIA scenarios and compared with state-of-the-art detection methods. Experimental results demonstrate that the proposed framework improves FDIA detection accuracy while maintaining a low false alarm rate, highlighting the feasibility of patient-specific digital twins for securing IoMT healthcare systems. The scope of this study is limited to the WUSTL-EHMS-2020 dataset, which contains continuous multi-sensor physiological telemetry collected from a single monitored subject. This setting is suitable for evaluating the proposed FDIA detection framework because it provides a consistent physiological baseline for modelling normal behaviour and enables the effects of stealthy data manipulation to be examined while preserving the temporal and spatial relationships among physiological sensors. Accordingly, the results demonstrate the feasibility of the proposed framework within the evaluated subject-specific setting, while generalisability across different patients is left for future work.

The remainder of this paper is organised as follows. Section 2 presents the related work, Section 3 describes the developed FDIA threat model, and Section 4 introduces the proposed detection framework. Section 5 presents the experimental setup and evaluation, while Section 6 discusses the results and analysis. Finally, Section 7 concludes the paper.

## 2. Related Work

FDIAs are one of the major threats to data integrity and security in cyber-physical systems. They have been widely studied in applications such as industrial control systems (ICSs), smart grids, power systems, and wireless sensor networks because they manipulate sensor measurements and disrupt normal system operation. Two main detection approaches dominate the literature. The first is model-based detection, which relies on state estimation and residual analysis using a known physical system model. The second is data-driven detection, which applies statistical methods, anomaly detection, or machine learning to identify abnormal temporal patterns and spatial relationships in the data.

Classical model-based detection methods such as those in [19,25,30,31,32,33,34] are vulnerable to stealthy and adversarial FDIAs. Liu et al. [25] showed that an attacker with knowledge of the system model can generate false measurements that satisfy the system constraints, allowing the attack to bypass residual-based detectors. It has been shown that coordinated stealthy FDIAs can evade conventional residual-based state estimation detectors by exploiting the mathematical properties of state estimation. To overcome this limitation, data-driven and machine learning methods such as those in [17,28,35,36,37] have been developed to learn normal system behaviour from historical data instead of relying only on physical models. Although these methods have improved FDIA detection in many cyber-physical systems, their application to healthcare remains challenging [1,9,11]. Unlike industrial systems, physiological data are generated by the human body and exhibit significant variability between patients [9]. Measurements that are normal for one patient may be abnormal for another. As a result, temporal and spatial relationships are patient-specific and should be modelled individually rather than using a population-based baseline [9]. This makes FDIA detection more difficult because malicious data can remain within the normal clinical range while still deviating from a patient’s true physiological pattern. If such attacks are not detected, they may affect clinical decision-making and compromise patient safety.

Recently, FDIAs have received increasing attention in IoMT applications. Several review studies have discussed the security risks of FDIAs in medical devices and IoMT systems, highlighting their impact on patient safety, data integrity, and healthcare service reliability, as well as the challenges of securing these environments [2,38,39,40,41,42,43]. However, despite the growing interest in this area, only a limited number of studies have proposed dedicated methods for detecting, preventing, or mitigating FDIAs in IoMT-based healthcare systems.

Ahmed and Barkat Ullah [42] presented one of the earliest studies focusing on False Data Injection Attacks (FDIAs) in healthcare environments. However, the study was primarily limited to raising awareness about the potential impact of FDIAs on healthcare systems and highlighting the necessity of developing preventive measures. Similarly, the survey in [41] investigated the integrity requirements of IoMT devices and identified the risks of data manipulation, highlighting the need for effective mechanisms to ensure the reliability and trustworthiness of healthcare data. Yaacoub et al. [2] reviewed the security and privacy issues of IoMT devices, including common attacks, existing countermeasures, and recommendations to overcome current limitations. The study analysed IoMT security vulnerabilities and attack surfaces, showing how cyber-attacks can compromise medical devices and communication channels, potentially enabling false data injection through the manipulation of healthcare data. Likewise, a study in [43] presented a systematic review of IoMT security threats and machine learning-based defence approaches. The study highlighted the potential of ML-based techniques for detecting malicious manipulation of healthcare data and improving the security of IoMT systems. While these studies provide valuable overviews, they focus on threat analysis and security challenges rather than developing FDIA detection methods. The study in [39] presented a cyber-attack taxonomy for IoMT and identified False Data Injection as a major integrity threat in IoMT. It also discussed adversarial attacks targeting ML-based healthcare systems, highlighting the security risks associated with intelligent IoMT applications. However, the work focused on threat classification and did not investigate detection methods for stealthy FDIAs.

Several studies have proposed machine learning-based detection models. Sankepally et al. [12] presented a detection framework for IoMT using an XGBoost classifier. However, the evaluation was limited to randomly generated false data injection scenarios and did not address stealthy attacks or adversarial manipulation designed to bypass ML-based detection models. The authors in [44] proposed an explainable ensemble-based intrusion detection system using Random Forest, Decision Tree, SVM, and XGBoost classifiers with Applying Shapley Additive Explanations (SHAP) for explanation. The WUSTL-EHMS-2020 dataset was used for training and validating the proposed IDS model. However, the study does not specifically investigate stealthy or adversarial FDIAs. The model mainly detects attack patterns based on network traffic features and does not evaluate whether adversaries can evade the classifier through adversarial manipulation of input features. Rani et al. [28] compared several machine learning algorithms, including K-Nearest Neighbours (KNNs), Random Forest (RF), Decision Tree (DT), and Artificial Neural Network (ANN), for detecting FDIAs. The ANN model achieved the highest detection accuracy compared with the other approaches. However, relying on a predefined input feature space and labelled datasets may create static attack patterns that limit the model’s ability to generalise evolving FDIA scenarios. In addition, temporal correlations, which are critical for identifying stealthy and adversarial FDIAs, were not considered in the proposed approach.

Other studies have investigated ensemble learning and deep learning approaches. A stacking ensemble learning model is proposed in [26] combining XGBoost, Random Forest, and ANN as base learners with XGBoost as the meta-classifier. However, the evaluation used a general cybersecurity dataset rather than healthcare data, where the attacks do not accurately represent physiological FDIA scenarios. Hasan et al. [10] proposed DSTAN-Med, a deep learning framework for detecting FDIAs in IoMT sensor streams. The study comprises three main components: Dual-channel Attention Mechanism, a residual 1D-CNN module for local temporal feature extraction, and a Physiological Plausibility Filter (PPF) to identify injected values that violate physiological constraints. However, the approach relies on physiological plausibility constraints and conventional FDIA simulations, which limits its effectiveness against stealthy and adversarial FDIAs that produce clinically plausible but malicious data patterns. 

Alternative approaches have also been explored. The study in [45] proposed a game-theoretic framework for analysing false data injection in personal e-health systems. The Age of Incorrect Information (AoII) metric [46] was used to calculate the average value of the age of incorrect information and derive Nash equilibrium strategies between legitimate users and adversaries. However, the study did not use real-world datasets and was evaluated only through numerical simulations based on predefined system parameters. Zachos et al. [27] proposed anomaly-based intrusion detection framework for IoMT networks. Several unsupervised and semi-supervised models were evaluated including One-Class SVM (OCSVM), Local Outlier Factor (LOF), Kernel Density Estimation (KDE), Gaussian Mixture Models (GMM), Minimum Covariance Determinant (MCD), and Isolation Forest. However, these methods were designed to detect anomalies in network traffic and device behaviour rather than manipulation of physiological measurements. Similarly, authors in [29] demonstrated that FDIAs can deceive wearable-device authentication systems, but the work focused on authentication rather than detecting manipulated physiological sensor data. The study [11] proposes a privacy-preserving framework for detecting FDIAs in AI–IoMT systems using distributed learning. However, such frameworks assume the availability of sufficient local experience of attack patterns, which may limit their effectiveness in detecting previously unseen or evolving FDIA strategies that differ from the learned attack behaviours.

In summary, existing studies demonstrate the potential of machine learning and anomaly detection methods for FDIA detection in IoMT systems. However, many existing approaches focus on network-level security threats, rely on predefined attack patterns, or use population-level models that do not consider individual physiological differences. Authors in [29] emphasise that detecting FDIAs in physiological data streams requires learning the individual’s personal signal signature, because the attack works precisely by staying close enough to the victim’s own data to avoid generic detectors. However, limited attention has been given to modelling patient-specific temporal and spatial relationships or detecting stealthy and adversarial FDIAs that maintain clinically plausible measurements while deviating from an individual’s normal behaviour. These limitations highlight the need for patient-specific detection frameworks capable of learning individual physiological dynamics, motivating the use of digital twin-based approaches for more robust FDIA detection in IoMT healthcare systems.

To address these limitations, this study proposes a patient-specific spatio-temporal FDIA detection framework for IoMT-based healthcare systems. The proposed framework uses a GCN–GRU digital twin to learn the individual patient’s physiological dynamics by modelling both spatial relationships between sensors and temporal dependencies in physiological signals. A Kalman filter-based shadow state is then used to generate innovation features that represent deviations between the expected and observed physiological states, enabling the detection of stealthy and adversarial FDIAs.

## 3. FDI Attack Model

Several studies have demonstrated how False Data Injection can bypass traditional detection mechanisms including the conventional Bad Data Detection (BDD) and Intrusion Detection Systems (IDSs) [47]. In IoMT environments, an adversary can generate stealthy attacks that evade detection by exploiting knowledge of physiological signal characteristics, device behaviour, sensor correlations, or the underlying detection systems. A comprehensive survey of FDIA techniques has been reviewed by Liang et al. [48]. In their survey, the authors demonstrated how stealthy and valid FDIAs can be constructed under various levels of attacker knowledge regarding network topology, detection mechanisms, and system configuration. In this study, it was demonstrated how stealthy and adversarial FIDAs manipulate measurement data within IoMT applications to inject consistent but false data in the system model.

To inject stealthy false data into a sensor stream, the attack must be introduced gradually rather than abruptly. If an attacker simply switches a sensor reading from its normal value to a manipulated value in a single step, the sudden change produces a large spike in the rate of change of the signal. Let us assume that the attack starts at a specific time in an attack campaign to either raise or hide an anomaly event to achieve attack objectives while becoming stealthily undetected. To simulate the stealthy behaviour, the concept of the raised-cosine envelope is introduced in this study. The envelope controls how much of the false data is mixed into the real sensor reading at each point in time. At the start of a campaign, the envelope value is zero, meaning the sensor reading is entirely unchanged. The envelope then rises smoothly to one over the onset period, during which the injected false data is gradually mixed in until the full attack magnitude is reached. It remains at one through the sustain period, where the attack is fully active. Finally, it falls smoothly back to zero over the offset period, removing the attack gradually rather than stopping it abruptly. The raised-cosine envelope $\epsilon \left(t\right)\in \left[0,1\right]$ has three stages: onset, sustain, and offset. The attack $\epsilon \left(t\right)$ is smoothly blended in and out, eliminating derivative spikes at campaign boundaries as follows:

$$\epsilon \left(t\right)=\left\{\begin{array}{c} \begin{array}{ccc} \frac{1}{2}\left(1-cos\left(\frac{\pi (t-s)}{\alpha L}\right)\right)\;\;\;\;\;\;\;\;\;\;\;\;\;\;\;\;\;\;\;\;\;\;\;\;\;\; & \;s\leq t\leq s+\alpha L & \left(onset\;phase\right) \end{array}\\ \begin{array}{ccc} 1\;\;\;\;\;\;\;\;\;\;\;\;\;\;\;\;\;\;\;\;\;\;\;\;\;\;\;\;\;\;\;\;\;\;\;\;\;\;\;\;\;\;\;\;\;\;\;\;\;\;\;\;\;\;\; & s+\alpha L\leq t\leq e-\beta L & \left(sustain\;phase\right) \end{array}\\ \begin{array}{ccc} \frac{1}{2}\left(1+cos\left(\frac{\pi (t-e+\beta L)}{\beta L}\right)\right)\;\;\;\;\;\;\;\;\;\;\;\;\;\; & e-\beta L\leq t\leq e & \left(offset\;phase\right) \end{array} \end{array}\right.$$

where s, e are the attack campaign interval [s, e] and l denotes to the length of the attack L=e−s. The parameters α and β represent the onset and offset fractions of the attack campaign, respectively, each expressed as a proportion of the total campaign length L. The onset phase occupies the first αL timesteps, during which the injected deviation is blended smoothly from zero to its full magnitude. The offset phase occupies the final βL timesteps, during which the deviation is returned smoothly to zero. The intervening period of length (1−α−β)L constitutes the sustain phase, during which the attack operates at full magnitude.

Let S be the targeted sensor index such that $S\subseteq \left\{1,\ldots ,N\right\},$ where N is the total number of sensors. The blended attack value for any targeted sensor jϵS, the target attack vector ${\hat{x}}_{t,j}$ at time t  against sensor j:

$${\hat{x}}_{t,j}\;=\left(1-\epsilon \left(t\right)\right)x_{t,j}\;+\epsilon (t)f_{t,j}$$

where $f_{t,j}$ is the attack-type-specific injected value defined below.

Based on this concept, five FDIA models are designed: Stealthy Drift (SD), Stealthy Bias (SB), Adversarial Biased Shadow (ABS), Spatial Inconsistent (SI), and Temporal Inconsistent (TI). These attacks are considered under two adversary capability levels. The model-agnostic attacks, namely SD, SB, SI, and TI, modify sensor measurements using physiological knowledge and sensor network topology without access to the defender’s prediction model. In contrast, the ABS attack represents a model-aware adversary that possesses a copy of the trained GCN–GRU digital twin and uses its predictions to generate injected measurements that minimise the resulting Kalman innovation, thereby directly targeting the residual-based detection mechanism. All five attacks use a unified raised-cosine envelope to introduce and remove the injected measurements gradually, avoiding abrupt changes at the attack boundaries. In this study, the term adversarial attack refers specifically to model-aware attacks that exploit knowledge of the trained GCN–GRU digital twin; gradient-based evasion attacks against the CNN-1D classifier are outside the scope of the present evaluation. All five attacks use a unified raised-cosine envelope to introduce and remove the injected measurements gradually, avoiding abrupt changes at the attack boundaries. The individual attack models are described below.

(1) Stealthy Drift Attack

The attacker gradually shifts the sensor reading by a bounded linear drift:

$$f_{t,j}=x_{t,j}\;+min(\gamma \left(t-s\right),{∆}_{max})$$

where γ is the drift rate and ${∆}_{max}$ denotes to maximum deviation, hence:

$${\hat{x}}_{t,j}=x_{t,j}+\epsilon \left(t\right)min(\gamma \left(t-s\right),{∆}_{max})\;$$

**Figure 1:**
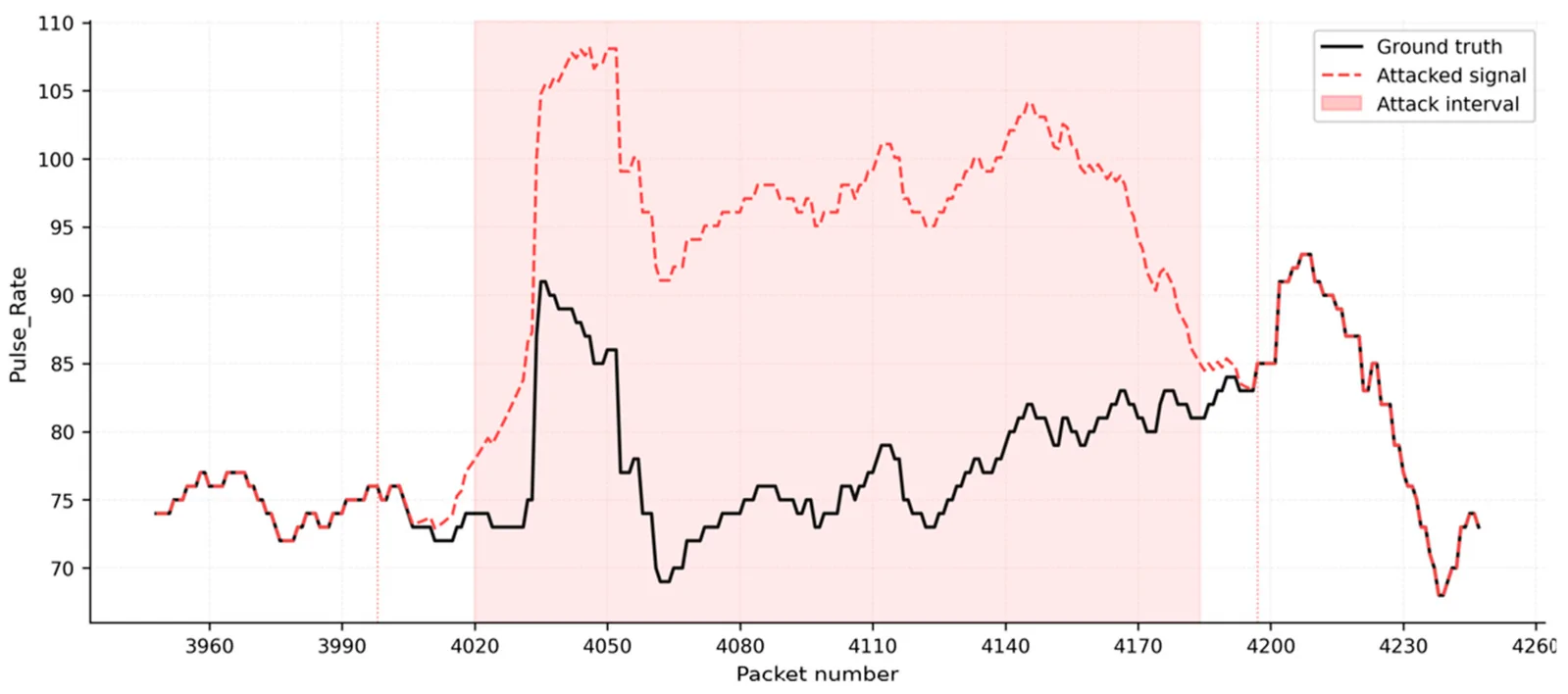
Stealthy drift attack.

As shown in Figure 1, the Pulse Rate sensor gradually drifts upward during the attack period. The attacked signal (red dashed line) separates from the ground truth (black line) as the deviation increases but remains within the clinical normal range of the individual user. Since the change occurs gradually, the attack is difficult to detect using instantaneous residual-based detectors.

(2) Stealthy Bias Attack

**Figure 2:**
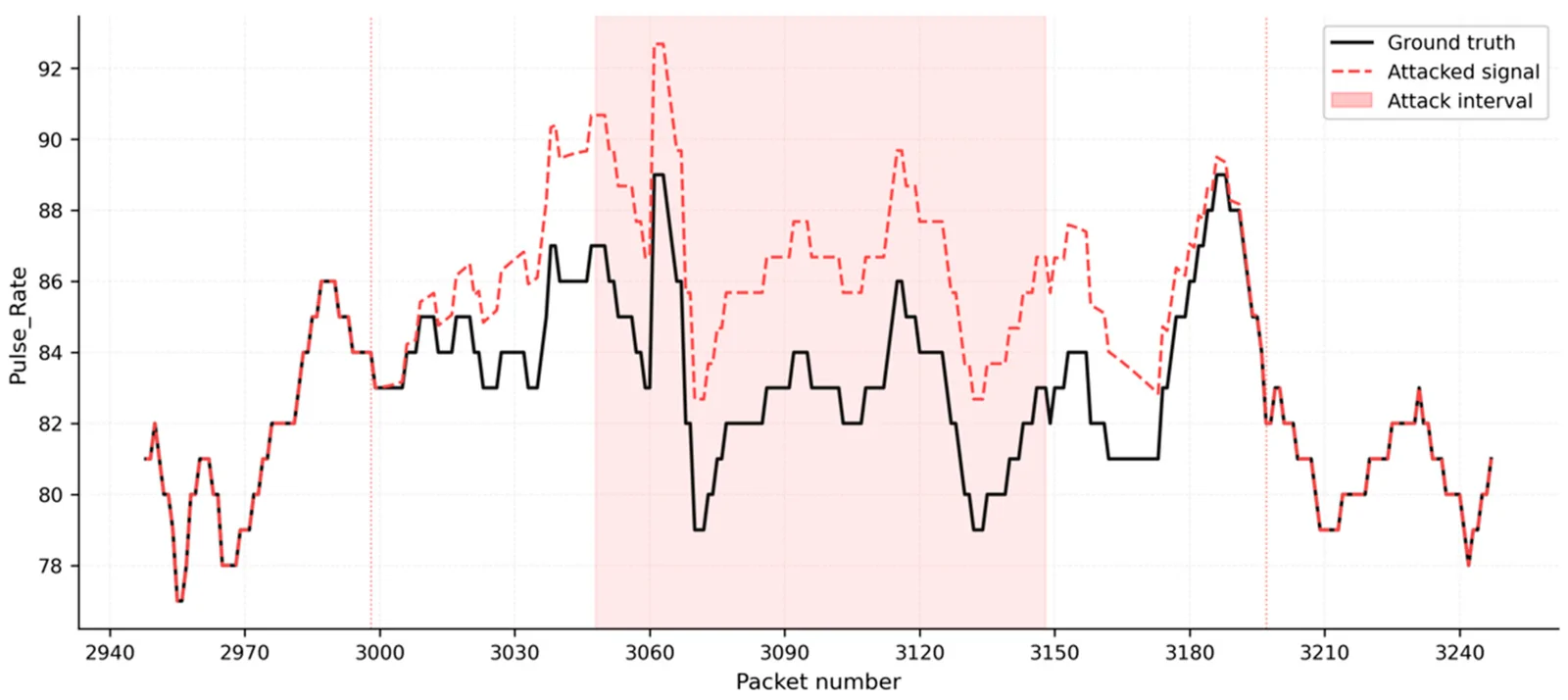
Stealthy bias attack.

In the Stealthy Bias Attack (e.g., in Figure 2), the attacker introduces a constant shift into the physiological measurements during an attack period. Instead of generating random abnormal values, the attacker adds a fixed offset to the original measurement, making the attack appear as a small but persistent deviation from the patient’s normal behaviour. The attacked measurement is defined as:

$${\hat{x}}_{t,j}=x_{t,j}+{\epsilon \left(t\right)∆}_{sb}$$

where $\Delta_{sb}$ denotes the stealthy bias and introduces a constant shift in the sensor measurement. Figure 2 depicts an example of stealthy biased attacks applied against Pulse_Rate sensor data with $\Delta_{b}$ = 0.5.

(3) Adversarial Biased Shadow

The Adversarial Biased Shadow (ABS) attack represents a strong model-aware adversary that possesses a copy of the trained GCN–GRU digital twin. The adversary uses the digital twin to obtain the predicted physiological state and constructs injected measurements that remain close to the expected patient-specific behaviour while introducing a controlled bias. To emulate realistic sensor compromise, a constant bias is added to the predicted state associated with the Kalman filter algorithm (the digital twin),

$$f_{t,j}={\overset{´}{x}}_{t,j}+{∆}_{ab}$$

where ${\overset{´}{x}}_{t,j}$ denotes the physiological state predicted by the GCN–GRU digital twin for sensor j, and ${∆}_{ab}$ denotes the adversarial bias. The resulting attacked measurement is then generated using the raised-cosine attack envelope:

$$\;{\hat{x}}_{t,j}=(1-\epsilon \left(t\right))x_{t,j}\;+\epsilon \left({\overset{´}{x}}_{t,j}\;+{∆}_{ab}\right)$$

**Figure 3:**
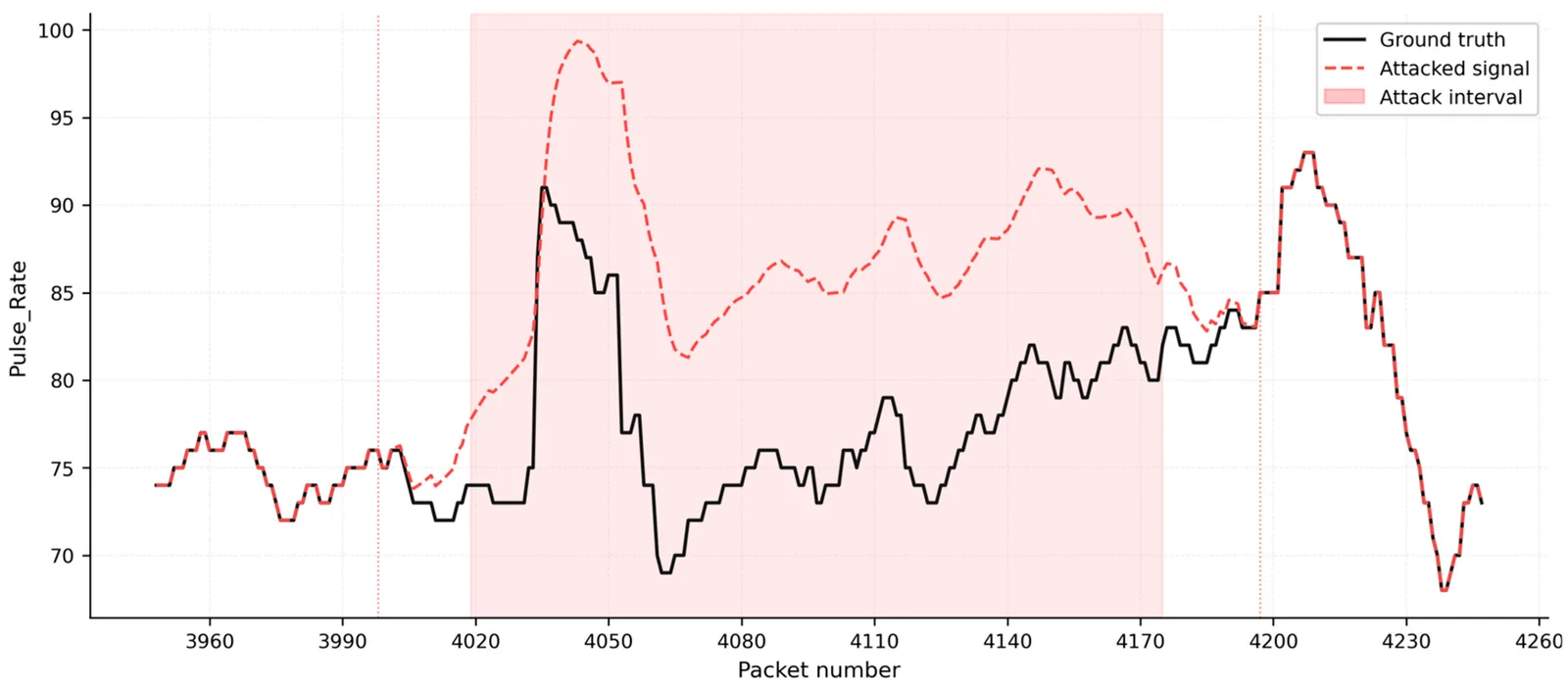
Adversarial biased shadow.

The attack is designed to remain close to the expected physiological state and reduce the resulting Kalman innovation, thereby challenging the innovation-based detection mechanism. Figure 3 depicts the adversarial biased shadow attack campaign. As shown in the Figure 3, the attacked signal deviates from the ground truth by a small but noticeable amount. The deviation is smaller than that produced by a conventional bias attack of the same magnitude because the shadow component partially compensates for the prediction error. The additional bias is introduced gradually and remains within a plausible physiological range, preventing simple threshold-based methods from easily identifying the abnormality.

(4) Spatial Inconsistency Attack

This attack intentionally violates physiological correlations while preserving each sensor’s temporal behaviour. The attack deliberately targets the sensor that is most embedded in the network. Thus, for every sensor j, compute its Pearson correlation $\rho_{jk}$ with every other sensor k, and count how many sensors satisfy $|\rho_{jk}|\geq \theta$ where θ is the correlation threshold.

$$j^{*}=\arg\;{max}_{j\;\in \left\{1,\ldots ,N\right\}}\sum_{k\neq j}1\;\left(|\rho_{jk}|\geq \theta \right)$$

Thus, the sensor with the largest number of correlated neighbours will be selected to be attacked. The chosen sensor is the most connected node in the physiological correlation graph. This makes it an ideal target because altering it while leaving its correlated neighbours unchanged creates the greatest disruption to the normal spatial relationships. The attacker applies stealthy drift to $j^{*}$ only; all correlated neighbours are left unchanged:

$${\hat{x}}_{t,j}=\left\{\begin{array}{c} x_{t,j^{*}}\;+\epsilon \left(t\right).\min\left(\gamma \left(t-s\right),{∆}_{max}\right)\;\;\;\;\;\;\;\;\;\;\;\;\;j=j^{*}\\ x_{t,j}\;\;\;\;\;\;\;\;\;\;\;\;\;\;\;\;\;\;\;\;\;\;\;\;\;\;\;\;\;\;\;\;\;\;\;\;\;\;\;\;\;\;\;\;\;\;\;\;\;\;\;\;\;\;\;\;\;\;\;\;\;\;\;\;\;\;j\neq j^{*} \end{array}\right.$$

Although the temporal evolution of the attacked sensor appears normal, the overall correlation structure between physiological variables becomes inconsistent.

**Figure 4:**
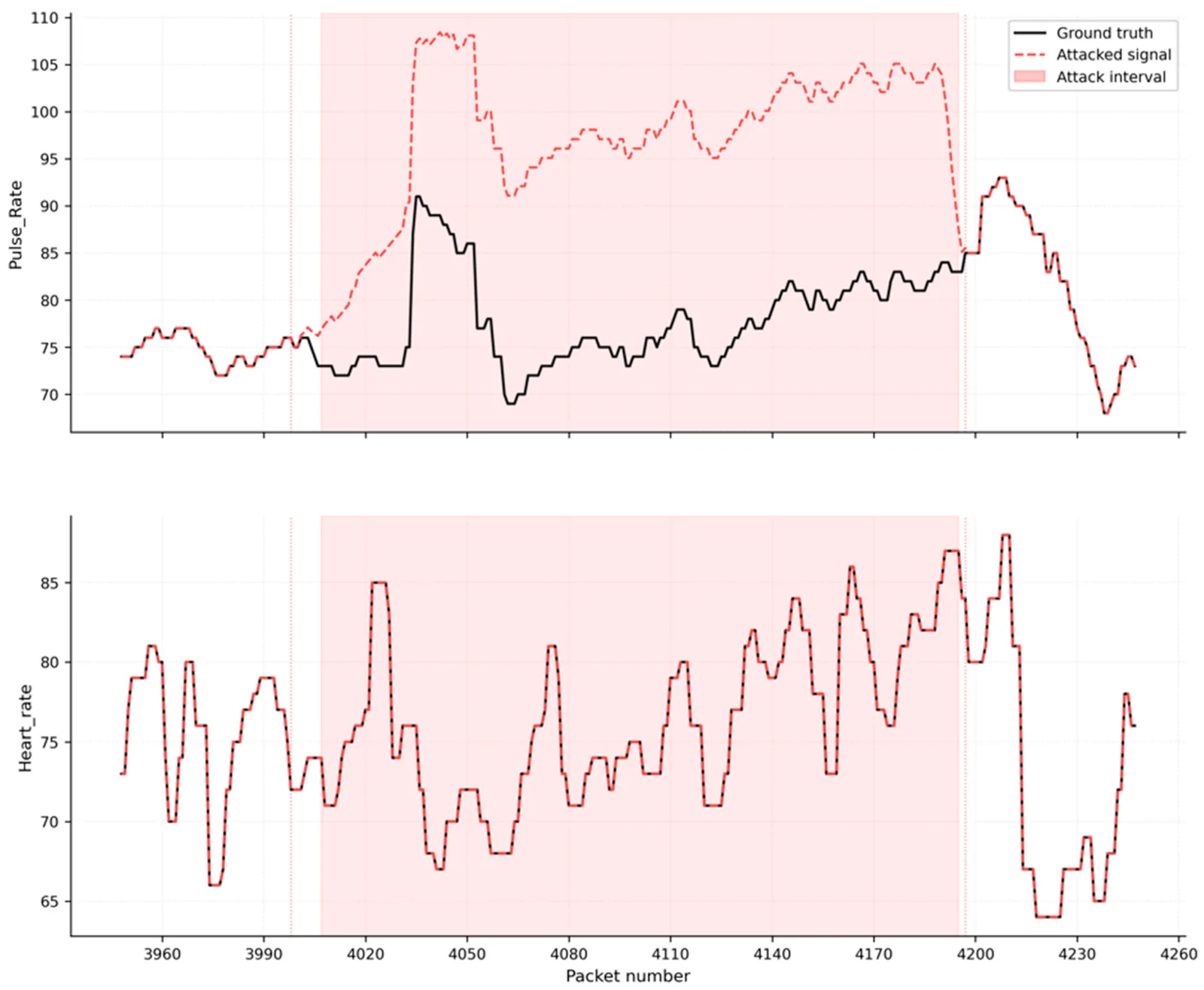
Spatial inconsistency attack.

Figure 4 shows example of Spatial Inconsistency Attack applied against the pulse rate sensors data. The top panel shows the Pulse Rate sensor gradually drifting upward during the attack interval, while the bottom panel shows the correlated Heart Rate sensor continuing its normal pattern without any change. The divergence between the two signals during the shaded attack period represents a spatial inconsistency. This inconsistency may remain undetected when each sensor is analysed independently but can be identified by monitoring the correlation between related physiological measurements.

(5) Temporal Inconsistent Attack Model

The temporal inconsistency attack targets the temporal dynamics of physiological signals rather than their spatial relationships. A cluster of highly correlated sensors is first identified using the physiological correlation graph. All sensors within the cluster are then shifted simultaneously by a coordinated step change,

$${\hat{x}}_{t,j}=x_{t,j}+\epsilon \left(t\right)d_{j}{∆}_{ti}\;$$

Because all correlated sensors are manipulated consistently, their instantaneous spatial relationships remain physiologically plausible.

**Figure 5:**
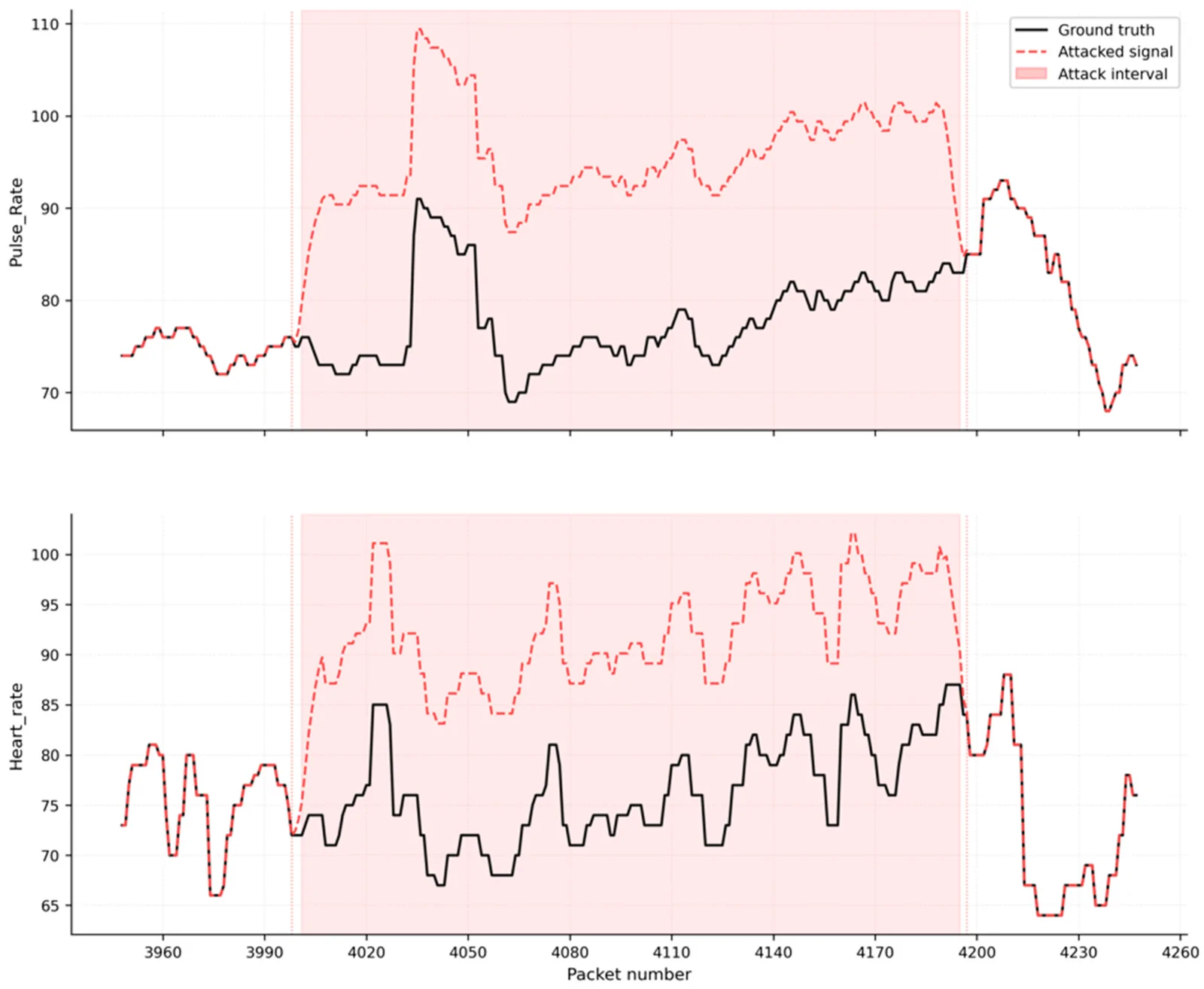
Temporal inconsistent attack model.

Figure 5 illustrates the Temporal Inconsistent Attack. As shown in the Figure 5, both Pulse Rate and Heart Rate increase simultaneously at the beginning of the attack interval. The two sensors are modified according to their normal correlation pattern, making the measurements appear physiologically plausible when analysed at an individual time point. This characteristic makes the attack challenging for existing detection approaches that rely on single-sensor analysis, predefined physiological thresholds, or spatial correlation checks, as the manipulated signals preserve their expected cross-sensor relationships.

## 4. Proposed FDIA Detection Framework

The proposed framework addresses the challenge of detecting stealthy FDIAs that exploit patient-specific physiological variations and evade existing generalised detection approaches by developing a patient-specific digital twin. The digital twin learns individual physiological behaviour and integrates spatio-temporal features with Kalman innovation analysis for attack detection. Although a fully informed adversary with complete access to the patient-specific digital twin could theoretically generate undetectable shadow attacks, such a scenario represents a complete system compromise beyond the capability of practical IoMT attackers. Therefore, this study focuses on realistic FDIA scenarios where attackers possess partial system knowledge and provides a robust and personalised detection mechanism for IoMT healthcare environments.

**Figure 6:**
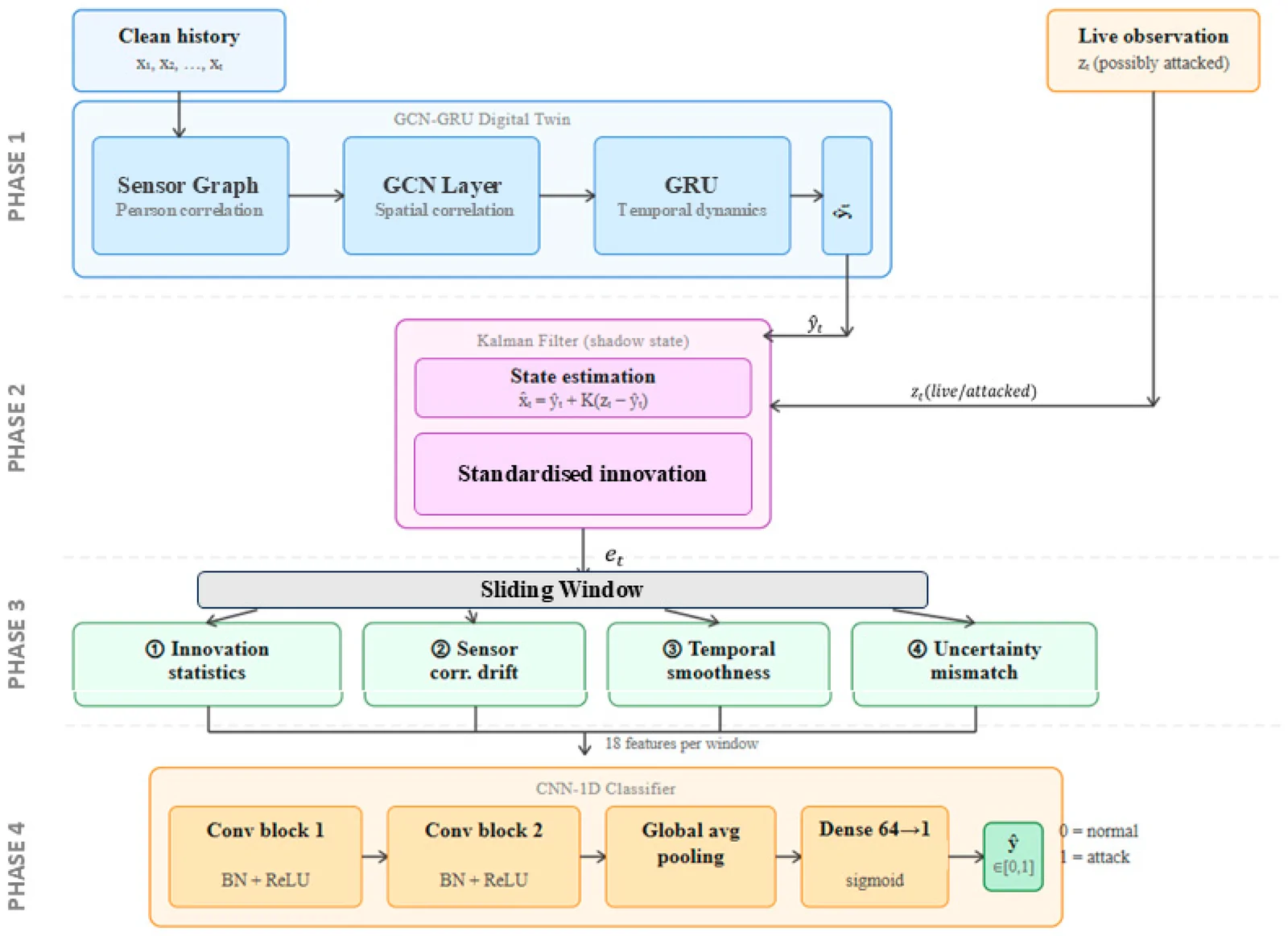
The proposed GCN–GRU(DT)-KF-CNN-1D detection framework.

The proposed detection framework consists of four sequential phases, as illustrated in Figure 6. Phase 1 describes the construction of the GCN–GRU digital twin for learning the spatial and temporal dependencies of physiological signals. Phase 2 presents the Kalman Filter-based shadow state estimation, where the predicted physiological state is compared with the observed measurements. Phase 3 describes the extraction of detection features using a sliding-window approach. Finally, Phase 4 presents the CNN-1D classifier.

Phase 1: GCN–GRU Digital Twin Construction

The digital twin is a spatio-temporal prediction model trained exclusively on the patient’s clean baseline physiological data. It predicts the expected next-step value of each sensor based on the previous observation window, while modelling both the temporal evolution of individual sensors and the spatial correlations among multiple sensors simultaneously.

Step 1: Sensor Graph Construction

A sensor graph $G=\left(V,E\right)$ is constructed from the patient’s baseline data, where each vertex v∈V represents a physiological sensor. An edge $\left(i,j\right)\in E$ is established when the Pearson correlation coefficient between sensors i and j exceeds a predefined threshold of 0.3. The symmetric normalised adjacency matrix is then computed as $A_{\mathrm{norm}}=D^{-1/2}\hat{A}D^{-1/2}$, where $\hat{A}=A+I$ represents the adjacency matrix with added self-loops (identity matrix) and D denotes the corresponding degree matrix. The normalised adjacency matrix is used as the input structure for the graph convolution operation.

Step 2: Graph Convolutional Layer Construction

The spatial relationship between sensors is represented by a normalised adjacency matrix $A_{\mathrm{norm}}$, constructed from the correlation between sensor measurements. At each timestep t, the GCN layer aggregates information from neighbouring sensors and transforms the sensor features into spatial embeddings, where each timestep is represented by a sensor feature vector $X_{t}.$ The latent spatial representation $S_{t}$ at timestep t generated by the GCN after graph convolution is expressed as follows:

$$S_{t}=A_{\mathrm{norm}}X_{t}W_{\mathrm{gcn}}+b_{\mathrm{gcn}}$$

where $X_{t}$ represents the input feature vector at timestep t. Specifically, $X_{t}$ contains the measurements from the sensors represented in the graph G. At each timestep t within the input window, the GCN layer computes a spatially enriched feature representation as:

$$H_{t}^{\left(l+1\right)}=ReLU\left(A_{\mathrm{norm}}H_{t}^{\left(l\right)}W_{\mathrm{gcn}}+b_{\mathrm{gcn}}\right)$$

where $H_{t}^{\left(l\right)}$ represents the node feature matrix at timestep t and GCN hidden layer l, and $W_{\mathrm{gcn}}$ and $b_{\mathrm{gcn}}$ are learnable weights and biases. The GCN layer aggregates information from each sensor and its correlated neighbours, enabling the model to capture spatial relationships among physiological variables. In this study, the GCN hidden dimension was set to 16. The resulting GCN features were then passed to the GRU component, with an input window size of ten time steps, to model temporal dependencies and capture the dynamic evolution of the physiological signals over time.

Step 3: Gated Recurrent Unit

To capture the temporal evolution of physiological signals, the GRU processes the sequence of graph-enriched features $H_{1},H_{2},\ldots ,H_{T}$ generated by the GCN layer over an input observation window of ten time steps. The sensor features are arranged such that a shared GRU model processes all sensors simultaneously, enabling it to learn the common temporal dynamics of physiological signals. In this study, the GRU uses a hidden-state dimension of 32 units. The hidden state is updated through the GRU gating mechanism, which controls how previous information and new observations are combined:

$$h_{t}=\left(1-z_{t}\right)⊙h_{t-1}+z_{t}⊙{\tilde{h}}_{t}$$

where $h_{t}$ represents the current hidden state, $z_{t}$ is the update gate, and ${\tilde{h}}_{t}$ is the candidate hidden representation. This mechanism enables the model to retain relevant historical information while adapting to new physiological changes. To improve prediction stability, a residual connection is introduced between the last observed measurement and the GRU output. Instead of directly predicting the absolute physiological value, the model learns the expected deviation from the previous observation. Thus, the final predicted physiological state ${\hat{x}}_{t+1}$ at the next timestep is therefore obtained as follows:

$${\hat{x}}_{t+1}=x_{t}+W_{\mathrm{out}}h_{t}+b_{\mathrm{out}}$$

where $x_{t}$ is the most recent observed physiological state, and $W_{\mathrm{out}}h_{t}+b_{\mathrm{out}}$ represents the predicted residual. $W_{\mathrm{out}}$ and $b_{\mathrm{out}}$ denotes the learned weight matrix and bias vector, respectively. $W_{\mathrm{out}}$ denotes the output-layer weight matrix, which maps the GRU hidden representation $h_{t}$ from the 32-dimensional hidden space to the required output dimension. $b_{\mathrm{out}}$ is the learned bias vector.

The proposed GCN–GRU digital twin was implemented using a single graph convolutional layer with 16 hidden units, followed by a single-layer GRU with 32 hidden units. The sensor graph was constructed using the Pearson correlation coefficient, with a correlation threshold of 0.3 and a symmetrically normalised adjacency matrix. The model was trained using input windows of ten time steps with a stride of one time step. Model parameters were initialised using Xavier (Glorot) initialisation, with biases initialised to zero. Training was performed using the Adam optimiser with a learning rate of 0.001, a batch size of 32, and 100 training epochs. A fixed random seed of 42 was used to improve reproducibility.

Phase 2: Kalman Filter integration

The Kalman filter is used to integrate the digital twin prediction with the live sensor observation and generate the estimated state and innovation-based detection features. The filter operates as a parallel shadow state, where the digital twin receives only the patient’s clean historical data rather than the potentially corrupted measurements. This design prevents the digital twin from adapting to attacked signals and allows the innovation term to reflect the deviation between the expected physiological behaviour and the observed measurement. At each timestep, the prediction produced by the GCN–GRU digital twin was treated as the prior state estimation, while the corresponding sensor measurement was used as the observation.

$${\hat{x}}_{t}^{-}=f_{GCN-GRU}({\hat{x}}_{t-1})$$

where ${\hat{x}}_{t}^{-}$ is the prior state estimation predicted using the GCN–GRU model given the measurement matrix ${\hat{x}}_{t-1}$. The prior error covariance of the predicted state was first updated as follows:

$$P_{t}^{-}=\;P_{t-1}+Q$$

where $P_{t}^{-}$ is the estimated prior error covariance and Q is the process noise covariance and it represents the uncertainty associated with the GCN–GRU. In this study, the Q is set to 0.001 due to the high confidence of the prediction model, GCN–GRU. The Kalman gain K is computed as follows:

$$K_{t}=\frac{P_{t}^{-}}{P_{t}^{-}+R}=\frac{P_{t-1}+Q}{P_{t-1}+Q+R}$$

where R denotes to measurement noise covariance which represents the uncertainty of the sensor measurements. During the attack campaign, KF should believe GCN–GRU more than sensor-measurements, thus Q << R. Accordingly, the measurement noise covariance estimated the variance of sensor measurements. The final state estimation ${\hat{x}}_{t}^{+}$ (the updated phase of Kalman filter) is computed as follows:

$${\hat{x}}_{t}^{+}=\;{\hat{x}}_{t}^{-}+\;K_{t}v_{t}$$

where $v_{t}$ denotes to the innovation error of Kalman filter. The innovation represents the discrepancy between the received measurement and the prior state prediction generated by the GCN–GRU model. It is defined as the difference between the observed measurement $y_{t}$ d and the prior state prediction ${\hat{x}}_{t}^{-}$. Thus, the innovation error of the Kalman filter is given by:

$$v_{t}=y_{t}-{\hat{x}}_{t}^{-}$$

where $y_{t}$ is the observed measurement and ${\hat{x}}_{t}^{-}$ denotes the prior state prediction generated by the GCN–GRU model. Under normal operating conditions, the innovation is expected to remain statistically consistent with its predicted uncertainty.

$$P_{t}^{+}=P_{t}^{-}+R$$

where $P_{t}^{+}$ postrior error covariance after the updated phase (the final state estimate). Deviations from this statistical behaviour may indicate measurement disturbances, sensor faults, or malicious modifications. However, stealthy false data injection attacks aim to generate manipulated measurements that preserve the statistical characteristics of the innovation, allowing them to bypass conventional residual-based detectors. Therefore, monitoring the innovation sequence and its normalised form provides an important mechanism for identifying abnormal measurement behaviour, while the Kalman update incorporates measurements according to their estimated uncertainty to achieve robust state estimation. However, adversarial and stealthy FDIAs attempt to conceal malicious modifications by generating measurements that remain within the expected error statistics, thereby reducing the apparent innovation. To reduce the risk of estimator drift caused by feedback of potentially compromised state estimates, the corrected state estimate from the Kalman measurement-update step ${\hat{x}}_{t-1}^{+}$ is not fed back into the prediction model, thereby preserving the sensitivity of subsequent innovations to attack-induced deviations. The received measurement $y_{t-1}$ is used as the basis for the subsequent prediction, while the estimated uncertainty is propagated through the filtering process. This design prevents malicious modifications from being accumulated through recursive state feedback, since the uncertainty propagation of the Kalman Filter remains independent of the estimated state trajectory. Consequently, an attacker attempting to introduce gradual measurement deviations must maintain consistency with both the observed sensor history and the learned spatio-temporal dynamics of the GCN–GRU model.

Phase 3: Feature Extraction

To capture attack-related deviations while reducing the impact of normal physiological variations, a sliding window of size 30 was applied to the standardised innovation vector $W=\{v_{1},\;v_{1},\;v_{1},\ldots ,v_{30}\}$. For each detection window, 18 features are calculated and organised into four complementary groups, as summarised in Table 1. Each set is designed to capture specific attack characteristics, including abnormal innovation behaviour, changes in sensor relationships, temporal inconsistencies, and prediction uncertainty variations. These features are designed to preserve short-duration attack signatures that may be lost when using simple averaging, as averaging can reduce the impact of attacks occurring only in a small number of samples within the window. A detailed description of the 18 features is provided below.

(1) Innovation Statistical Features: These features describe the magnitude, distribution, and temporal trend of the innovation values. These features are designed to capture attacks that introduce gradual or persistent measurement deviations. This includes peak innovation ($f_{1}=max(\left|W\right|)$), mean deviation ($f_{2}=mean(\left|W\right|)$), innovation variability ($f_{3}=std(\left|W\right|)$), drift trend ($f_{4}=slope\;(\left|W\right|)$), cumulative deviation $f_{5}=\sum max(0,\;P(W\leq P_{90})$, and the proportion of abnormal measurements $f_{6}=count(\left|W\right|>P_{75th})/count(W)$. The peak innovation feature $f_{1}$ is highly discriminative for abrupt or high-amplitude attacks because significant deviations from the digital twin prediction result in large innovation values. Compared with the peak innovation, the mean deviation feature $f_{2}$ is less influenced by isolated outliers and is therefore more sensitive to sustained low-level deviations characteristic of stealthy attacks. The innovation variability $f_{3}$ is the standard deviation of the absolute innovation sequence. Persistent bias attacks increase both the mean and variability of the innovations, while adversarial shadow attacks generate distinctive variance patterns that differ from those produced by normal measurement noise. The drift trend feature $f_{4}$ is obtained by fitting a first-order linear regression to the sequence of maximum absolute signal values within each (W)-sample sliding window. The resulting slope coefficient quantifies gradual changes in signal magnitude and enables the detection of stealthy drift attacks. A positive slope indicates progressively increasing innovations, a characteristic signature of stealthy drift attacks, whereas a near-zero slope is more indicative of constant bias or normal system behaviour. The cumulative deviation, $f_{5},$ measures the accumulated magnitude of deviations that exceed the 90th percentile of the data. This statistic accumulates evidence of persistent deviations that individually remain below instantaneous detection thresholds, making it particularly effective for detecting gradual drift attacks. The proportion of abnormal measurements $f_{6}$, quantifies the fraction of measurements exceeding the 75th percentile of the data. Persistent attacks affecting multiple sensors or extended time periods significantly increase this proportion. These features were designed to effectively detect attacks that produce persistent or gradually increasing deviations, such as stealthy bias and drift attacks.

(2) Sensor Correlation Drift Features: These features measure changes in the spatial relationships between sensors by comparing the current window correlation structure with the normal correlation pattern. They help identify attacks that disrupt physiological dependencies between correlated sensors. Features in this group include overall correlation drift, the Frobenius distance ($f_{7}=\left\|(C_{w}-\;C_{norm}\right\|$), where $C_{w}$ is the correlation matrix, graph correlation drift $\left(f_{8}=\sum_{\left(i,j\right)\in E}\left|C_{w\left(i,j\right)}-\;C_{norm\left(i,j\right)}\right|/\left|E\right|\right)$, where E represents graph edges, and maximum correlation difference $\left(f_{9}=\max\left|C_{w}-\;C_{norm}\right|\right)$. The overall correlation drift $f_{7}$ is the Frobenius norm between the current $C_{w}$ and reference correlation matrices $C_{norm}$. This feature summarises overall changes in pairwise sensor relationships and is sensitive to localised disruptions affecting only a subset of sensor pairs. The graph correlation drift $f_{8}$ is the mean absolute correlation difference computed only for sensor pairs connected within the physiological sensor graph. Restricting the calculation to strongly correlated sensor pairs improve sensitivity to attacks that selectively violate expected physiological relationships while reducing sensitivity to unrelated measurement noise. The maximum correlation difference $f_{9}$ is the largest absolute correlation difference among all sensor pairs. Unlike the previous two features, this feature emphasises the most severely affected sensor relationship, enabling reliable detection even when only a single pair exhibits abnormal behaviour. These features are specifically designed to detect attacks that manipulate individual sensors while preserving the remaining measurements, thereby disrupting expected inter-sensor correlations.

(3) Temporal Smoothness Features: These features quantify abrupt changes and irregular temporal behaviour in the innovation sequence, including maximum first-order derivative $\left(f_{10}=\max(W_{t}-W_{t-1}\right)$, mean first derivative $f_{11}=\mathrm{mean}(W_{t}-W_{t-1})$, maximum second-order derivative $f_{12}=\max(W_{t}-{2W}_{t-1}+W_{t-2})$, and number of abrupt changes above the mean $f_{13}=\mathrm{count}\left(\left|W_{t}-W_{t-1}\right|>1.5std(\right))$. The maximum first-order derivative $f_{10}$ is the largest absolute first-order difference between consecutive innovation samples across all sensors. The mean first derivative $f_{11}$ is the average absolute first-order difference throughout the detection window. This feature complements the maximum jump by detecting sustained fluctuations rather than isolated discontinuities. The $f_{12}$ is the maximum absolute second-order difference of the innovation sequence. This feature measures rapid changes in temporal dynamics and is particularly sensitive to abrupt attack onset and offset transitions. The $f_{13}$ is the number of innovation differences exceeding a threshold of 1.5 standard deviations. Whereas the maximum jump identifies the largest discontinuity, this feature quantifies the frequency of significant temporal changes, distinguishing systematic attacks from isolated noise spikes. These features are designed to detect attacks that preserve spatial consistency but introduce abnormal temporal transitions.

(4) Uncertainty Mismatch Features: These features evaluate the consistency between observed innovations and the Kalman filter uncertainty model using innovation energy (the mean Chi-Squared statistic) and autocorrelation measures, including the mean square value of the innovation sequence $f_{14}=\sum W^{2}/count(W)$, the deviation innovation error energy $f_{15}=std(\sum W^{2}/count(W)),$ maximum innovation error energy $f_{16}=max(\sum W^{2}/count(W))$, the mean autocorrelation $f_{17}=mean\left(corr\left(W_{t},W_{t-1}\right)\right),$ and the maximum autocorrelation $f_{18}=max(corr\left(W_{t},W_{t-1}\right))$. The mean square value of the innovation sequence $f_{14}$ is the average per-sensor squared standardised innovation across the detection window. Since squared standardised innovations follow a chi-square distribution under nominal conditions, values greater than one indicate degraded filter consistency caused by persistent attacks. The deviation innovation error energy $f_{15}$ is the standard deviation per-sensor chi-squared statistic. The maximum innovation error energy $f_{16}$ is the largest per-sensor chi-squared statistic. This feature highlights the most anomalous sensor and is particularly effective for identifying attacks targeting a single measurement channel. The mean autocorrelation $f_{17}$ is the average lag-1 autocorrelation of the innovation sequence across all sensors. Persistent attacks introduce temporal dependence into the innovations, violating the Kalman filter whiteness assumption and increasing autocorrelation. The maximum autocorrelation $f_{18}$ is the largest lag-1 autocorrelation among all sensors. By focusing on the most affected channel, this feature improves sensitivity to attacks involving only a small subset of sensors, where averaging across all channels may obscure anomalous behaviour. These features identify deviations from the expected statistical properties of normal filter residuals.

These 18 extracted features provide complementary information about attack magnitude, spatial inconsistency, temporal irregularity, and uncertainty deviation, enabling robust detection of diverse FDIA scenarios.

Phase 4: CNN-1D Classifier

**Figure 7:**
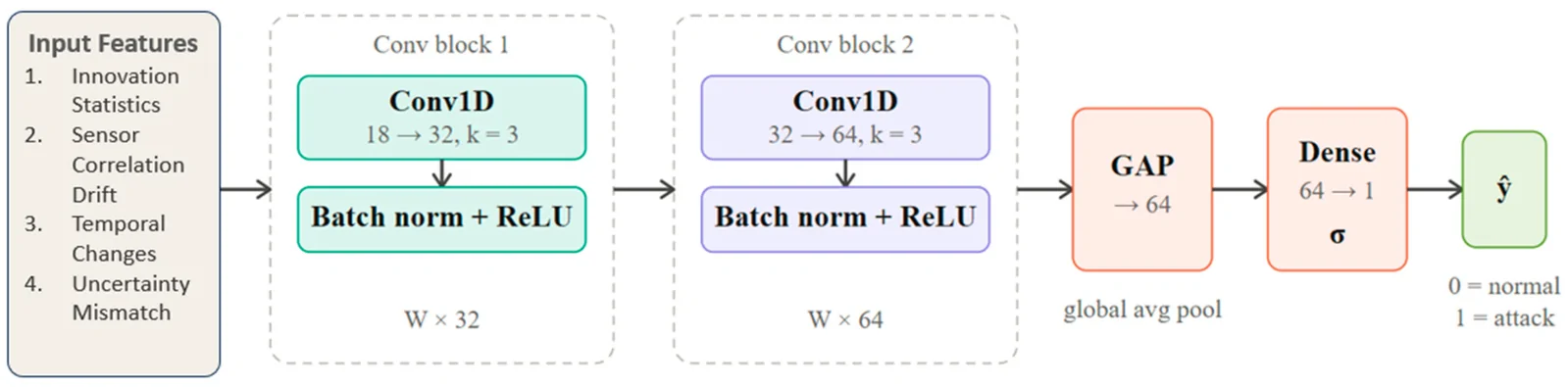
The proposed CNN-1D architecture.

The final phase employs a one-dimensional convolutional neural network (CNN-1D) to classify normal and attacked states based on the feature sequences extracted in Phase 3. The CNN-1D is designed to learn local temporal patterns and distinguish normal innovation behaviour from attack-induced anomalies. The CNN-1D classifier operates on sequences of 18-dimensional feature vectors extracted from overlapping detection windows. Although each window produces a single feature vector, consecutive windows overlap by (W − 1 = 29) samples, resulting in a continuous temporal sequence of feature representations. Figure 7 illustrates the complete forward pass of the CNN-1D classifier from left to right. The input consists of a sequence of feature vectors generated from the previous feature extraction phase (one-dimension input features size of 18). The CNN-1D architecture was selected for three main reasons. First, one-dimensional convolutions preserve the temporal ordering of consecutive feature vectors, allowing the network to capture local temporal patterns that are indicative of evolving attack behaviour. Second, parameter sharing reduces the number of trainable weights compared with a fully connected architecture, thereby mitigating overfitting on relatively small, labelled datasets. Third, global average pooling provides translation invariance by ensuring that anomaly detection is insensitive to the precise temporal position of an attack within the input sequence.

As shown in Figure 7, the proposed CNN-1D classifier consists of two convolutional blocks, followed by global average pooling and a fully connected output layer. The first block applies a one-dimensional convolution with 18 input channels, 32 output channels, and a kernel size of three. The convolution captures short-term temporal dependencies among consecutive feature vectors while preserving their temporal ordering. Batch normalisation is subsequently applied to improve training stability, followed by a rectified linear unit (ReLU) activation to introduce non-linearity. The resulting feature representation has dimensions ((L,32)), where (L) denotes the input sequence length. The second convolutional block increases the representational capacity by mapping 32 input channels to 64 output channels using the same kernel size. This layer learns higher-level temporal patterns formed by combinations of lower-level features extracted in the previous phase. Batch normalisation and ReLU activation are again applied, producing an output of dimensions (L,64).

A global average pooling layer aggregates each of the 64 feature maps across the temporal dimension, producing a fixed-length 64-dimensional feature vector regardless of the input sequence length. This operation reduces the number of trainable parameters while making the learned representation less sensitive to the temporal location of anomaly patterns within the observation window, which is particularly beneficial for false data injection attacks whose abnormal patterns may occur at various positions within a window. The resulting 64-dimensional feature vector is then passed to a fully connected layer with a sigmoid activation function σ to estimate the probability $\hat{y}$ that the corresponding observation window contains a false data injection attack:

$$\hat{y}=\sigma \left(Wh+b\right)$$

where h is the feature vector produced by the global average pooling layer, W is the weight vector, b  is the bias, and $\hat{y}\in [0,1]$ represents the estimated probability that the observation window contains a false data injection attack. The global average pooling operation provides robustness against variations in the position of anomaly patterns within the observation window, which is particularly beneficial for gradual attacks where the maximum deviation may occur at different time positions. The lightweight CNN-1D architecture is designed to support potential deployment in resource-constrained environments.

## 5. Experimental Setup and Evaluation

The evaluation procedure is presented in the following subsections: Dataset Acquisition, Dataset Preparation, GCN–GRU Digital Twin Model Training, Attacks Implementation, CNN-1D Classifier Training, and Performance Metrics.

(1) Dataset Acquisition

The WUSTL-EHMS-2020 dataset [49] is used for evaluation. The dataset has been used by many researchers [50,51,52]. It contains 16,318 samples of continuous multi-sensor physiological telemetry collected from a subject-worn IoMT monitoring system. The dataset includes eight physiological sensor channels: skin temperature (Temp), blood oxygen saturation (SpO2), pulse rate (Pulse_Rate), systolic blood pressure (SYS), diastolic blood pressure (DIA), heart rate (Heart_rate), respiratory rate (Resp_Rate), and ST segment deviation (ST). The packet sequence number (Packet_num) is used as the temporal index to maintain the chronological order of measurements during preprocessing and window generation.

(2) Dataset Preparation

The raw dataset is inspected for data quality issues, including missing values, duplicate packets, and physiologically invalid measurements. Duplicate records are removed, while invalid sensor readings are treated as missing values to preserve the continuity of the time series. Missing values are then estimated using linear interpolation.

To reduce measurement noise while maintaining the natural variation of physiological signals, a moving-average filter is applied to each sensor channel. The data are then sorted according to the packet sequence number to ensure correct temporal ordering. Finally, Z-score normalisation is performed independently for each sensor channel to transform measurements into a common scale. A sliding window with a window length of (W = 10) and a stride of one time step was employed to construct the input sequences for the proposed digital twin GCN–GRU model. Each input instance consists of 10 consecutive time steps, while the target is the one-step-ahead observation immediately following the input window. Because the stride is one time step, consecutive windows overlap by nine time steps, allowing the model to capture temporal correlations in the physiological signals. This formulation enables the GCN–GRU to learn both the spatial dependencies among sensors and the temporal dynamics of historical physiological observations, thereby facilitating accurate prediction of future physiological states. The resulting pre-processed dataset is used as the input for the following phases of the proposed framework.

(3) GCN–GRU Digital Twin Model Construction

To train and validate the proposed GCN–GRU digital twin, a block-interleaved chronological split is used to learn the spatial and temporal correlations in the physiological data. After creating the sliding windows, the data is divided into 20 continuous chronological blocks. Each block is chronologically ordered. Using a fixed random seed, 70% of the blocks were selected for training and the remaining 30% for evaluation. Since the sliding window uses a stride of one time step, overlapping observations between the training and evaluation sets were removed from the evaluation subset to prevent information leakage. This approach maintains the time order within each block while providing a more representative evaluation set that includes different physiological conditions and signal variations. It reduces the risk of biased evaluation caused by selecting a single continuous section of the time series.

(4) Attacks Implementation

A total of 40 attack campaigns were generated, comprising eight campaigns for each of the five attack types. For each attack type, five campaigns were assigned to the training split, resulting in 25 training campaigns, while three campaigns were assigned to the evaluation split, resulting in 15 evaluation campaigns. Attack campaigns were injected into the pre-processed, z-score-normalised physiological sensor time series as temporally contiguous segments. Campaign start positions were sampled uniformly at random within the evaluation portion of the dataset, while campaign durations were sampled uniformly from the interval of 30–80 rows. A minimum coverage constraint was imposed to ensure that at least one campaign of each attack type was present in both the training and evaluation splits, thereby guaranteeing representation of all attack classes during model training and performance evaluation.

To avoid unrealistic signal discontinuities at campaign boundaries, which would trivially expose attack onset to rate-of-change detectors and artificially inflate detection performance, a raised-cosine blending envelope, ε(t) ∈ [0,1], was applied to all attack types (as explained in Section 3). The envelope controls the interpolation between the clean sensor measurement and the attack-specific injected value at each time step, generating the attacked observation as a weighted combination of the original and injected measurements. The envelope increases smoothly from zero to one over an onset fraction α of the campaign duration, remains at one during the sustain phase, and decreases smoothly back to zero over an offset fraction β. The cosine formulation ensures that the first derivative of the envelope is zero at both transition boundaries, eliminating the boundary spikes that would otherwise be introduced by linear interpolation.

For the attacks (Attacks 1 to 4), namely stealthy drift (Attack 1), stealthy bias (Attack 2), adversarial shadow (Attack 3), adversarial biased (Attack 4), and Spatial Inconsistent (SI), α = β = 0.25, meaning that 25% of each campaign was allocated to the onset transition, 50% to sustained full-magnitude injection, and 25% to the offset transition. In contrast, the temporal-inconsistent attack (Attack 5) used α = β = 0.05 to approximate an abrupt step transition, as temporal discontinuity is an inherent characteristic of this attack mechanism and excessive smoothing would alter its intended behaviour.

Ground-truth labels were defined based on effective signal deviation rather than the existence of numerical manipulation alone. Although measurements may be modified throughout an attack campaign, deviations below $\tau_{dev}\;=\;0.5\sigma$ normalised units were considered indistinguishable from normal physiological variability and were therefore assigned negative labels. This prevents the detector from being penalised for failing to identify perturbations that fall within the expected operating range of the sensor signals. Positive labels were assigned only when the injected deviation exceeded the normal variability boundary and represented an observable attack condition. Consequently, the CNN-1D classifier is not trained using positive labels associated with perturbations that fall within the expected range of physiological variability and measurement uncertainty. Such deviations are considered indistinguishable from normal sensor fluctuations and are therefore treated as non-actionable observations for the detection task. Campaign-span labelling was evaluated and discarded because it assigned positive labels to transition regions where the injected deviation remained within the acceptable noise boundary, resulting in ambiguous training samples. Approximately 25–30% of campaign-labelled positive samples exhibited no measurable deviation beyond the predefined detection threshold.

Two adversary capability levels were considered. Model-agnostic attacks (Attacks 1, 2, 4, and 5) modified sensor measurements using only physiological knowledge and sensor-network topology, without access to the defender’s prediction model. Model-aware attacks (Attack 3) assumed that the adversary possessed a copy of the trained GCN–GRU digital twin and exploited this model to generate injected values that minimise the resulting Kalman innovation, thereby directly targeting the residual-based detection mechanism.

All attack-specific parameters, including the drift rate γ = 0.06σ/row, drift limit $\;\Delta_{max}=\;3.0\sigma$, stealthy bias magnitude $\Delta_{sb}\;=\;2.0\sigma$, adversarial bias offset $\Delta_{ab}\;=\;1.5\sigma$, temporal step magnitude $\Delta_{ti}\;=\;2.5\sigma$, and deviation threshold $\tau_{dev}=0.5\sigma$ were fixed across all experiments. These magnitudes were defined in standard-deviation units σ based on the empirical statistics of the 14,268 clean observations retained after preprocessing, providing data-driven perturbation constraints rather than formally validated clinical reference ranges. Campaign locations and target sensor assignments were generated using a fixed random seed of seven. The complete attack configuration is provided in Table 2.

Because the WUSTL-EHMS-2020 dataset represents a single subject, physiological plausibility in this study is defined relative to the subject-specific empirical distribution of the clean observations rather than population-level clinical reference intervals. The attack magnitudes therefore represent controlled deviations from the subject’s observed physiological baseline and are not intended to constitute clinically validated pathological trajectories. Formal clinical expert validation of the generated attack trajectories was beyond the scope of this study and is identified as future work.

(5) CNN-1D Classifier Construction

The CNN-1D architecture employs two convolutional layers with 32 and 64 output channels, respectively, each using a kernel size of three and ReLU activation. Batch normalisation follows every convolutional layer to improve optimisation stability, while global average pooling replaces conventional fully connected hidden layers to reduce model complexity and improve generalisation. The network is trained using the Adam optimiser with an initial learning rate of 0.001 for 300 epochs.

The CNN-1D classifier is intentionally separated from the GCN–GRU digital twin. The digital twin is trained exclusively on normal operating data to predict physiological measurements, whereas the CNN-1D is trained using labelled attack data to classify anomalous innovation patterns derived from the prediction residuals. This modular design allows each component to be optimised independently for its respective regression and classification tasks, thereby improving the interpretability and robustness of the overall detection framework.

The same block-interleaved chronological partition is used for all experimental conditions. The 2848 detection windows are divided into twenty equal-length contiguous temporal blocks, with twelve blocks (1708 windows) assigned to training and eight blocks (1140 windows) reserved for evaluation. This partition avoids random temporal mixing and ensures that both subsets contain representative mixtures of active and quiet physiological periods. The evaluation subset is not used during classifier training or threshold optimisation.

(6) Performance Metrics:

The performance of the proposed model is evaluated using six standard classification metrics: Accuracy, Precision, Detection Rate (Recall), False Alarm Rate (FAR), F1-score, and Area Under the Receiver Operating Characteristic Curve (AUC-ROC). These metrics are commonly used in related studies to evaluate the performance of False Data Injection Attack (FDIA) detection systems. Accuracy measures overall classification correctness, while Precision and Recall quantify the reliability and completeness of attack detection, respectively. FAR is particularly important in healthcare monitoring because excessive false alarms can reduce the practical usability of the detection system. F1-score provides a balanced measure of Precision and Recall, particularly under class imbalance, whereas AUC-ROC assesses the model’s discriminative capability across different decision thresholds. Collectively, these metrics provide a comprehensive assessment of detection effectiveness and operational reliability.

## 6. Results Analysis and Discussion

This section presents the experimental results, including the performance of the digital twin, FDIA detection results, comparisons with baseline methods, and detection performance across different attack types. The experiments are designed to assess the proposed framework from complementary perspectives. First, the predictive capability of the GCN–GRU digital twin is evaluated against a naive persistence predictor to assess its ability to model normal physiological dynamics. Second, the contribution of the digital-twin and Kalman innovation features is examined by comparing multiple classifiers using the same feature set with and without the digital twin. Third, the proposed framework is compared with commonly used statistical, unsupervised, and reconstruction-based FDIA detection methods under the same experimental setting. Finally, detection performance is analysed for individual attack types to assess the framework’s effectiveness across different FDIA behaviours. This evaluation design enables the contributions of the physiological prediction model, innovation-based features, and classification architecture to be assessed separately while maintaining a consistent basis for comparison.

The evaluation is structured around three complementary evaluations. First, it evaluates the contribution of the GCN–GRU digital twin by comparing the proposed classifiers under WITH-DT (with the digital twin shadow state) and WITHOUT-DT (without the digital twin shadow state) conditions. Second, it compares the proposed classifiers with five baseline methods using features extracted from the GCN–GRU digital twin innovations. Third, it examines detection performance for each attack type to identify the strengths and limitations of the evaluated methods.

**Figure 8:**
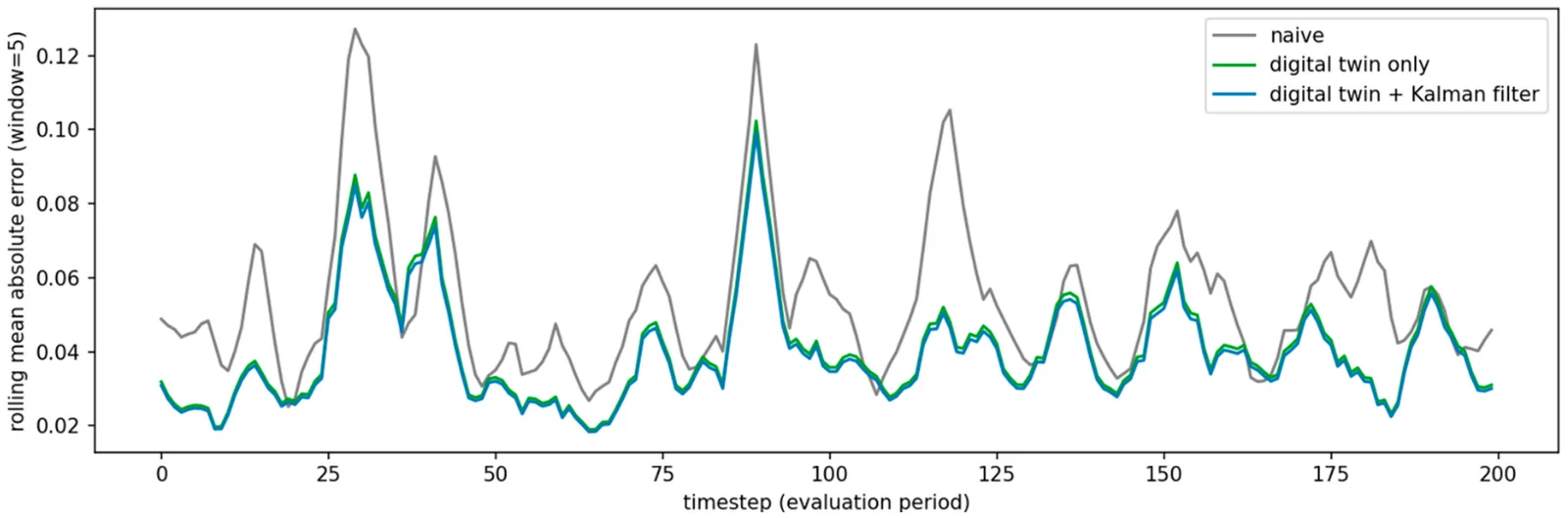
Prediction error comparison in terms of (MAE).

The GCN–GRU digital twin is evaluated on the held-out evaluation set and compared with two baselines: a naive persistence predictor, which assumes no change from the last observed value, and the GCN–GRU digital twin combined with Kalman filter fusion. The naive persistence model provides a strong baseline for slowly varying physiological signals. The digital twin achieves a mean absolute error (MAE) of 0.0363, representing a 7.4% improvement over the naive baseline (MAE = 0.0392). Incorporating Kalman filter fusion further reduces the MAE to 0.0315, corresponding to a 19.6% improvement over the naive persistence model. Figure 8 depicts the smoothed prediction error for the three studied models.

**Figure 9:**
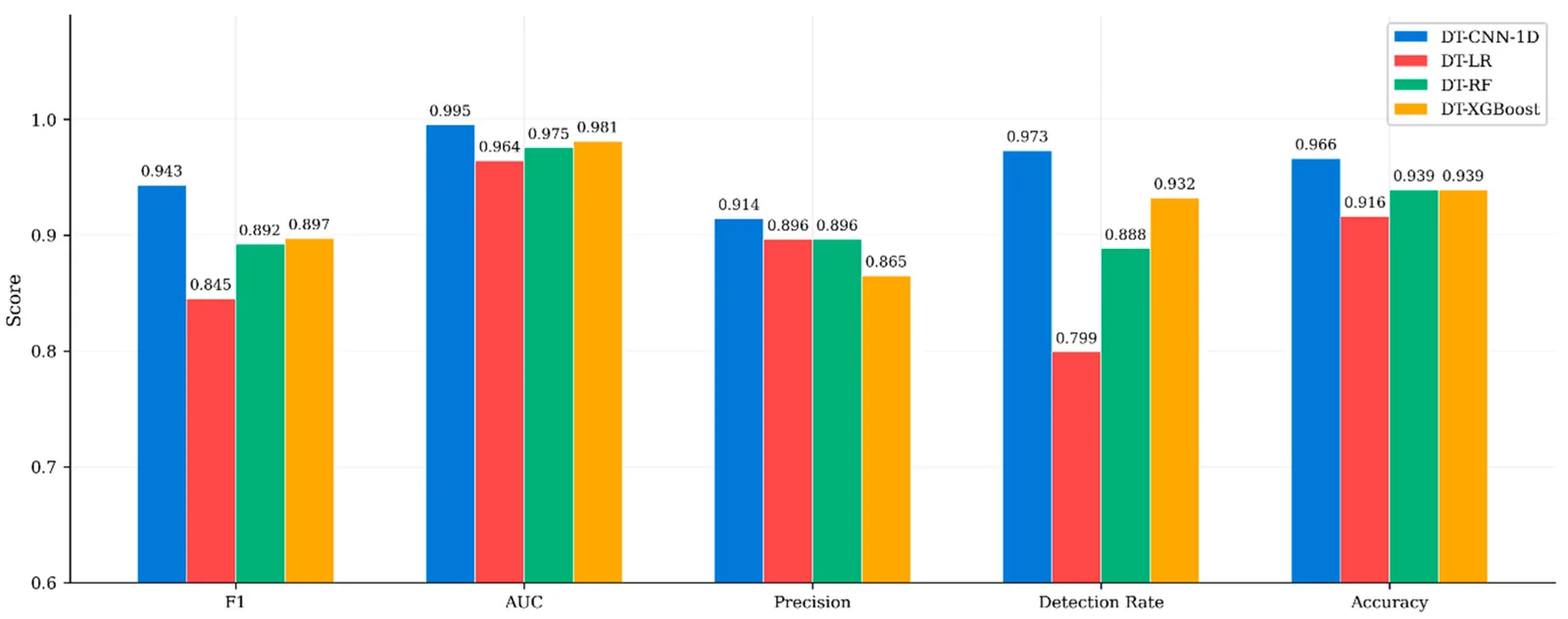
Performance comparison of proposed classifiers using digital-twin- and Kalman-derived features.

Figure 9 presents the detection performance of the proposed DT-CNN-1D and three tested classifiers, namely DT-XGBoost, DT-RF, and DT-LR, all trained using the same window-based feature extraction as extracted from the proposed digital twin integrated with the Kalman filter. These classifiers are evaluated to investigate the effectiveness of the extracted digital twin-based features and to justify the selection of CNN-1D as the final detection model. As shown in Figure 9 and Table 3, DT-CNN-1D achieves the highest performance, with an F1-score of 94.3% and an AUC of 99.5%, followed by DT-XGBoost (F1 = 90.9%, AUC = 98.4%), DT-RF (F1 = 88.3%, AUC = 97.9%), and DT-LR (F1 = 86.0%, AUC = 95.9%). These results confirm that the digital twin and Kalman innovation features provide the main discriminative information for FDIA detection. DT-CNN-1D achieves superior performance because it can learn local temporal patterns within the innovation windows through convolutional operations. Unlike conventional classifiers that process extracted statistics as independent features, CNN-1D can capture sequential relationships among attack-induced deviations, making it more suitable for identifying temporal FDIA patterns.

**Figure 10:**
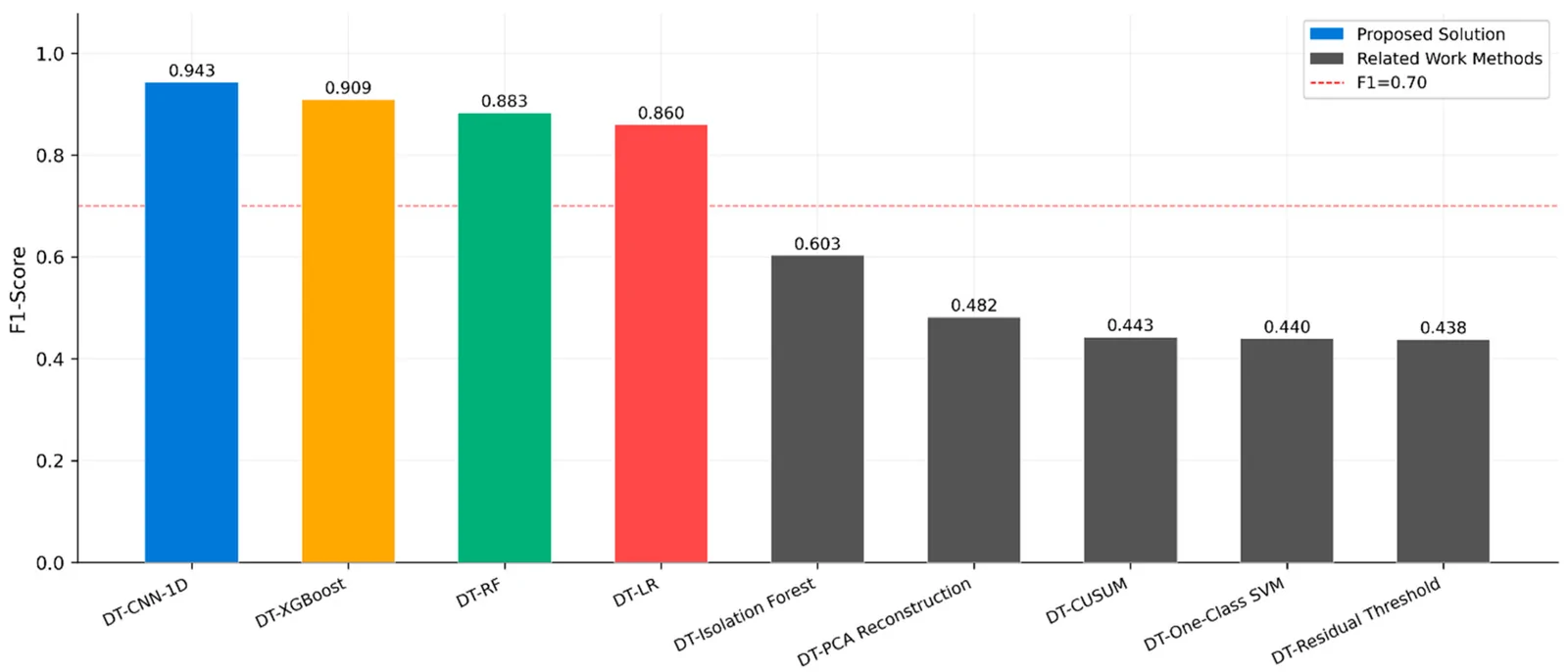
Overall accuracy performance comparison between proposed solution and related work.

Figure 10 compares the F1-score of the four proposed methods with five commonly used baseline detection methods, all evaluated using the Digital Twin (DT) shadow-state features. The related work methods evaluated in this study represent commonly reported approaches for false data detection, including statistical change detection (CUSUM), residual thresholding, unsupervised anomaly detection (Isolation Forest and One-Class SVM), and reconstruction-based detection (PCA Reconstruction) [16,53,54]. As shown in the Figure 10 and Table 3, the proposed DT-CNN-1D achieves the highest performance, followed by DT-XGBoost, DT-RF, and DT-LR. In contrast, the best-performing baseline method, DT-Isolation Forest, achieves an F1-score of only 60.3%, which is 34.0% lower than DT-CNN-1D. The baseline methods rely on unsupervised learning or statistical thresholds, which limits their ability to learn specific attack patterns from labelled data. In comparison, the proposed supervised classifiers can learn the temporal and cross-sensor characteristics of different FDIA behaviours. These results demonstrate that the digital twin provides an effective feature representation, while supervised learning is essential for achieving accurate and reliable detection for stealthy and adversarial FDIA.

**Figure 11:**
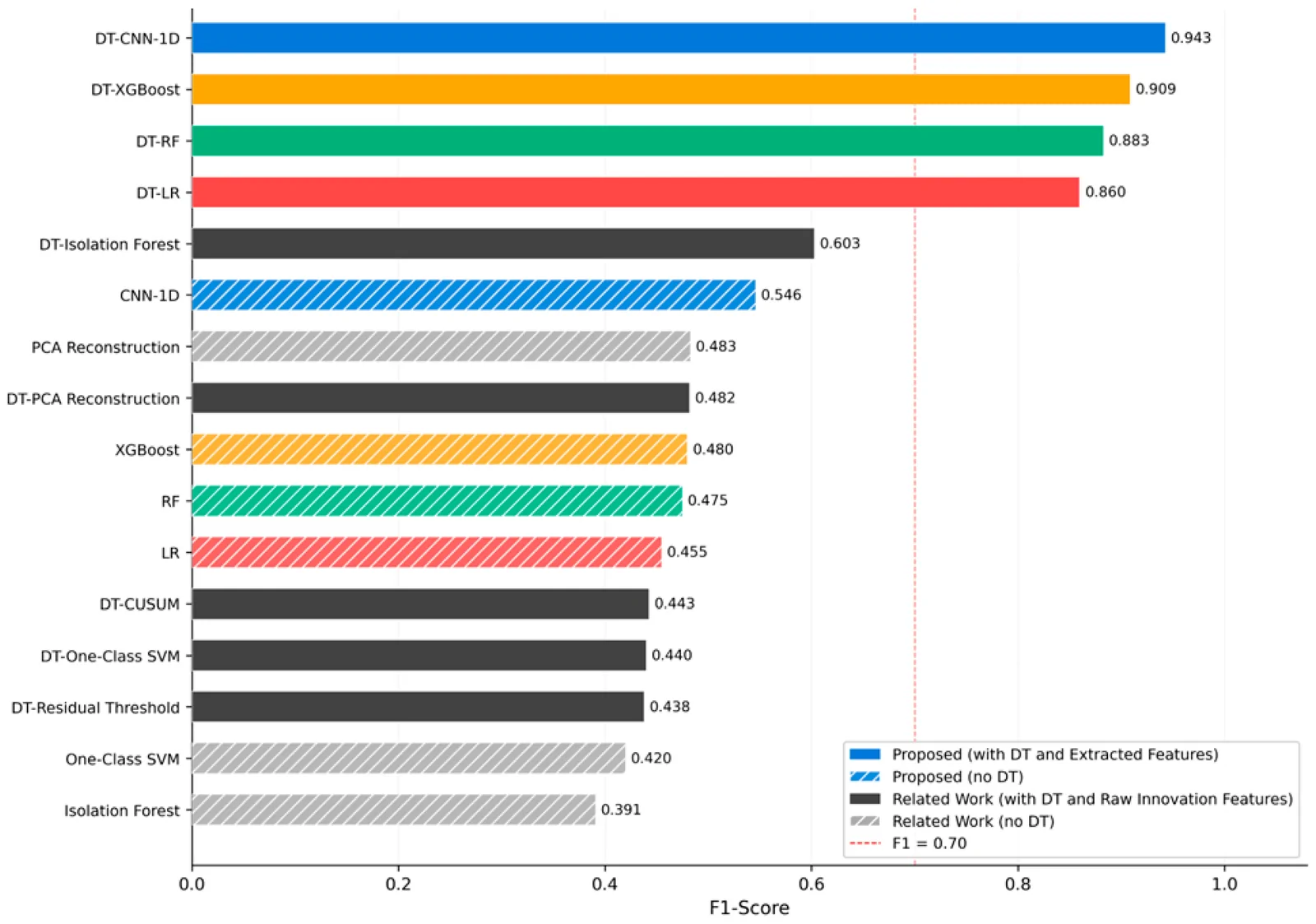
Overall performance (F1-Score) comparison between proposed solution and related work with and without the digital twin.

Figure 11 and Table 3 present the performance comparison across sixteen experimental configurations, comprising four proposed classifiers and five baseline methods. The proposed classifiers, namely DT-CNN-1D, DT-XGBoost, DT-RF, and DT-LR, were evaluated under both WITH-DT (with the digital twin shadow state) and WITHOUT-DT (without the digital twin shadow state) configurations, using the corresponding 18 window-based features. Similarly, PCA Reconstruction, Isolation Forest, and One-Class SVM were evaluated with and without the digital twin, using the standardised Kalman innovation errors directly rather than the 18 derived features. In contrast, DT-CUSUM and DT-Residual Threshold were evaluated only under the WITH-DT configuration using the same standardised Kalman innovation errors. This design enables the contribution of the digital twin to be assessed while comparing the proposed feature-based classifiers with established detection methods operating directly on the innovation errors.

As shown in Figure 11 and Table 3, the WITHOUT-DT configuration, in which features are extracted directly from the normalised raw sensor observations, produces substantially lower detection performance across all four proposed classifiers. CNN-1D achieves the highest F1-score of 54.6%, while the AUC values range from 67.9% to 74.6%. In contrast, using the DT shadow state and the corresponding Kalman innovation features substantially improves both F1-score and AUC across all classifiers. F1-score increases from 54.6% to 94.3% for CNN-1D, from 48.0% to 90.9% for XGBoost, from 47.5% to 88.3% for RF, and from 45.5% to 86.0% for LR. The improvement across different classifier architectures indicates that the performance gain is primarily associated with the more informative representation provided by the GCN–GRU digital twin and Kalman innovation features, rather than being specific to the CNN-1D classifier. These results support the role of the digital twin as a patient-specific shadow state for generating a more discriminative representation of deviations from the observed physiological behaviour.

As shown in Figure 11 and Table 3, the baseline results provide further insight into how the digital-twin-derived innovation representation contributes to FDIA detection. Among the baseline methods, DT-Isolation Forest achieves the highest F1-score of 60.3%, compared with 0.390 for Isolation Forest without the digital twin. This improvement indicates that the GCN–GRU shadow state produces an innovation representation that can enhance the detection of anomalous behaviour. However, this benefit is not consistent across all conventional detectors. The F1-score of PCA Reconstruction remains almost unchanged between the WITHOUT-DT and WITH-DT configurations (48.3% and 48.2%, respectively), while One-Class SVM shows only a limited improvement from 42.0% to 44.0%. DT-CUSUM and DT-Residual also achieve relatively low F1-scores of 44.3% and 43.8%, respectively, despite operating on the digital-twin-generated innovations. These results indicate that the digital twin provides informative deviation signals, but that direct processing of the innovation errors is insufficient to fully capture the complex characteristics of the stealthy and adversarial attacks considered in this study.

In contrast, the proposed classifiers use the same digital twin/Kalman innovation signal after applying the 18 window-based features designed to capture statistical, spatial, temporal, and uncertainty-related deviations. Their substantially higher F1-scores, ranging from 86.0% for DT-LR to 94.3% for DT-CNN-1D, together with substantially lower FARs, ranging from 5.9% for DT-LR to 3.6% for DT-CNN-1D, indicate that the proposed feature representation enables the discriminative information in the innovation sequence to be exploited more effectively. Thus, the results suggest that the detection improvement arises not from the digital twin alone, but from the integration of the patient-specific shadow state, Kalman innovation analysis, structured feature extraction, and supervised classification.

**Figure 12:**
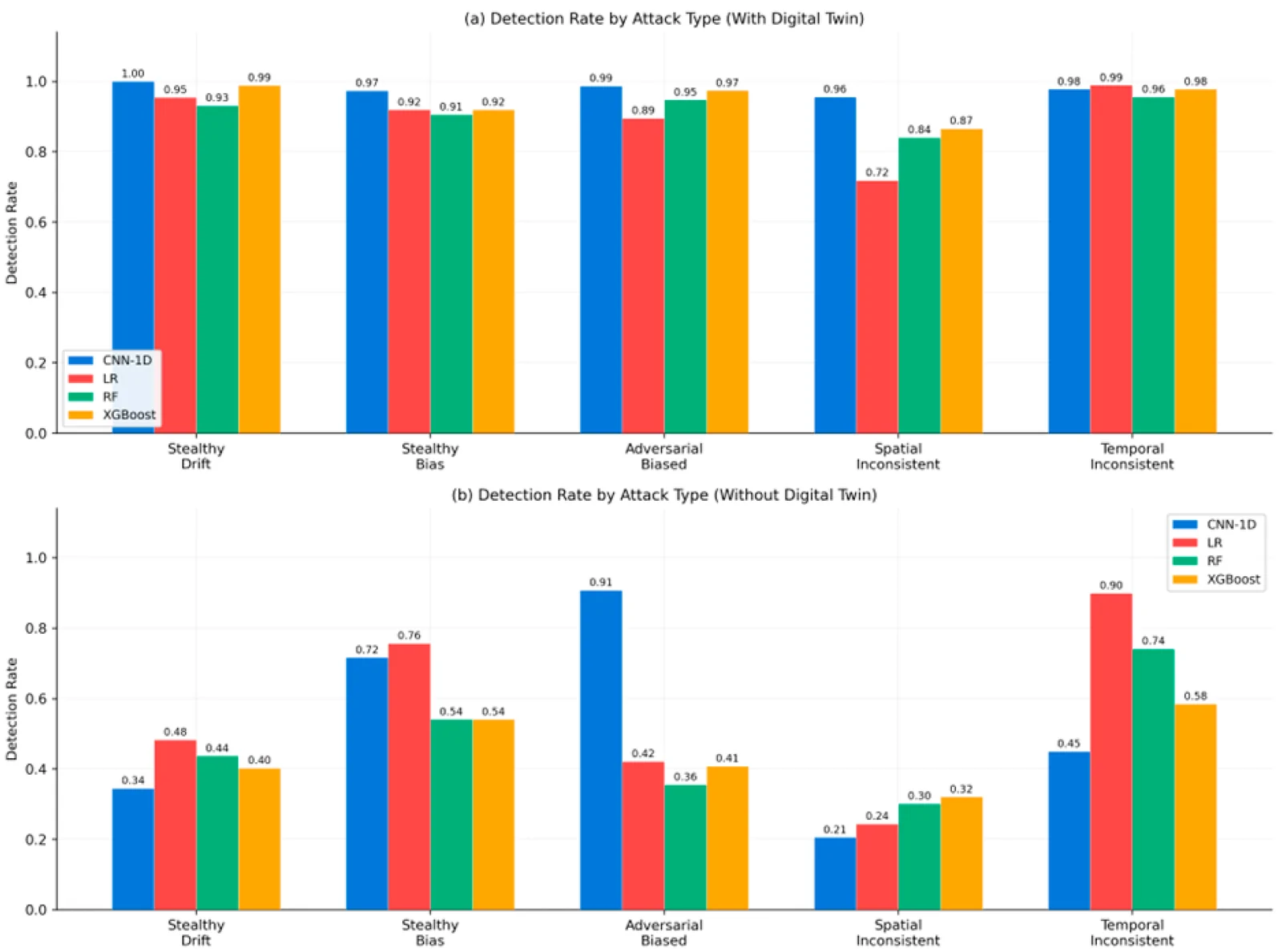
Detection Rate (**a**) WITH Digital Twin and (**b**) WITHOUT Digital Twin.

Figure 12 presents the detection rates for each attack type, revealing performance differences that are not captured by the overall evaluation metrics. Spatial-inconsistent attacks represent the most challenging scenario, with DT-CNN-1D achieving the highest detection rate (96%), while most baseline methods perform poorly due to their limited ability to capture changes in cross-sensor relationships. Stealthy drift attacks are also challenging without the digital twin; however, detection performance improves significantly across all proposed models when the digital twin is introduced with the extracted sets of features as discussed in Section 4 Phase 3. Adversarial biased shadow attacks are consistently detected by the proposed digital twin-based models, whereas detection performance decreases noticeably without the digital twin. Temporal-inconsistent attacks achieve high detection rates across most methods, indicating that their abrupt temporal changes are easier to identify.

The evaluation leads to four main findings. First, the GCN–GRU digital twin, together with the Kalman innovation analysis and window-based feature extraction, is the primary contributor to the improved detection performance, as removing the digital twin results in a substantial reduction in F1-score across all classifiers. Second, supervised classifiers trained on the extracted innovation-based features consistently outperform statistical and unsupervised baseline methods, achieving higher detection performance with lower false alarm rates. Third, the spatial-inconsistent attack results demonstrate the importance of modelling physiological sensor relationships, as methods without explicit spatial representation show limited capability in detecting changes in cross-sensor dependencies. Finally, DT-CNN-1D achieves the best overall performance, obtaining the highest F1-score and AUC, which confirms the effectiveness of integrating patient-specific GCN–GRU digital twin modelling, Kalman innovation-based feature extraction, and temporal pattern learning for FDIA detection in IoMT systems.

The proposed CNN-1D is lightweight by design, owing to its shallow architecture, limited channel widths, and global average pooling, which substantially reduces the number of trainable parameters and memory requirements. For the eight-channel physiological sensing system, the trained model contains approximately 8225 parameters, corresponding to approximately 32 KB when stored using 32-bit floating-point weights. This compact model footprint indicates its potential suitability for resource-constrained IoMT edge environments. However, hardware-level evaluation of inference latency, throughput, memory utilisation, and energy consumption was beyond the scope of the present study and will be investigated in future work using representative IoMT and edge-computing platforms.

## 7. Conclusions

This study presented a patient-specific spatio-temporal FDIA detection framework for IoMT-based healthcare systems. The proposed framework addresses key limitations of existing FDIA detection methods, which often rely on population-level models, predefined attack patterns, or detection mechanisms developed for non-healthcare cyber-physical systems. The framework integrates a GCN–GRU digital twin as a patient-specific shadow state, Kalman innovation analysis, window-based statistical feature extraction, and a CNN-1D-based detection model to identify anomalous physiological behaviours.

The evaluation demonstrates that the GCN–GRU digital twin and the derived innovation-based features make a substantial contribution to detection performance. Replacing the digital twin-generated features with raw sensor observations results in a substantial reduction in F1-score across all evaluated classifiers which confirms the importance of learning patient-specific spatio-temporal physiological patterns. The proposed DT-CNN-1D achieves an F1-score of 94.3% with a false alarm rate of 3.6%, which outperforms the evaluated baseline methods under the same feature conditions. These results demonstrate the feasibility of the proposed framework for detecting the evaluated stealthy and adversarial FDIA scenarios.

Despite these promising results, the main limitation of this study is the use of a single-patient dataset and synthetic attack scenarios, which limits the generalisability of the findings across diverse patient populations and real-world attack behaviours. Future work will focus on validating the framework using multi-patient clinical datasets and more diverse and realistic attack scenarios. A further limitation is the absence of systematic feature-ablation and sensitivity analyses to fully assess the contribution and robustness of the proposed feature representation under different configurations and attack conditions. Future studies will address these aspects to provide a more comprehensive evaluation of the framework. Further extensions will include the development of adaptive online digital twins, learned sensor graph structures, and federated learning to support continuous adaptation, improved modelling of sensor relationships, and privacy-preserving deployment.

## Figures and Tables

**Table 1:** Summary of the 18 derived features used for attack detection.

Feature	Description
Group 1	Innovation Statistical Features
$f_{1},f_{2},f_{3}\;$	These features are the maximum, average, and the standard deviation absolute innovation within the detection window, respectively.
$f_{4}$	The slope of a linear regression fitted to the maximum innovation magnitude at each timestep.
$f_{5}$	The final cumulative sum statistic computed using innovations exceeding the 90th percentile of data.
$f_{6}$	The proportion of innovation samples whose absolute value exceeds two standard deviations.
Group 2	Sensor Correlation Drift Features
$f_{7}$	The Frobenius norm between the current and reference correlation matrices.
$f_{8}$	The mean absolute correlation difference computed only for sensor pairs connected within the physiological sensor graph.
$f_{9}$	The largest absolute correlation difference among all sensor pairs.
Group 3	Temporal Smoothness Features
$f_{10}$	The largest absolute first-order difference between consecutive innovation samples across all sensors.
$f_{11}$	The average absolute first-order difference throughout the detection window.
$f_{12}$	The maximum absolute second-order difference of the innovation sequence.
$f_{13}$	The number of innovation differences exceeding a threshold of 1.5 standard deviations.
Group 4	Uncertainty Mismatch Features
$f_{14},\;f_{15},\;f_{16}$	The average, std, and largest per-sensor squared standardised innovation across the detection window, respectively.
$f_{17,\;}f_{18}$	The average and largest lag-1 autocorrelation of the innovation sequence across all sensors, respectively.

**Table 2:** Attack model configuration parameters.

Symbol	Value	Description
α	0.25	α denotes the onset fraction (Attacks 1–4), which is the proportion of campaign used for ramp-up
	0.05	Onset fraction (Attacks 5)
β	0.25	β denotes the offset fraction (Attacks 1–4), which is the proportion of campaign used for ramp-down
	0.05	Offset fraction (Attacks 5)
γ	0.06σ	γ denotes the drift slope, which is the rate of linear drift (σ/row)
$\Delta_{max}$	3.0σ	$\Delta_{max}$ is the drift cap, which is the maximum drift magnitude (σ)
$\Delta_{sb}$	2.0σ	Stealthy bias magnitude, which is a constant bias offset (σ)
$\Delta_{ab}$	1.5σ	Adversarial bias offset, which is a bias added to shadow prediction (σ)
$\Delta_{ti}$	2.5σ	$\Delta_{ti}$ is the temporal step magnitude, which is the coordinated step size (σ)
$\tau_{dev}$	0.5σ	Deviation label threshold: minimum deviation for positive label (σ)
θ	0.3	Graph threshold: minimum correlation for graph edge
−	7	Random seed, fixed at seven for all campaign sampling.

**Table 3:** Performance proposed solution and related work with and without the digital twin.

Method	F1	AUC	Recall	FAR	Precision	Accuracy
DT-CNN-1D	0.943	0.995	0.973	0.036	0.914	0.966
CNN-1D	0.546	0.746	0.464	0.093	0.665	0.781
DT-LR	0.86	0.959	0.867	0.059	0.853	0.92
LR	0.455	0.679	0.516	0.298	0.408	0.649
DT-RF	0.883	0.979	0.903	0.056	0.865	0.932
RF	0.475	0.72	0.451	0.178	0.502	0.716
DT-XGBoost	0.909	0.984	0.932	0.047	0.888	0.947
XGBoost	0.48	0.718	0.431	0.145	0.542	0.734
DT-Residual Threshold	0.438	0.382	0.981	0.995	0.282	0.283
DT-CUSUM	0.443	0.481	1	1	0.285	0.285
DT-Isolation Forest	0.603	0.778	0.812	0.351	0.479	0.695
DT-One-Class SVM	0.44	0.807	0.317	0.049	0.718	0.77
DT-PCA Reconstruction	0.482	0.791	0.391	0.092	0.628	0.761
Isolation Forest	0.391	0.564	0.458	0.352	0.341	0.594
One-Class SVM	0.42	0.605	0.592	0.49	0.325	0.534
PCA Reconstruction	0.483	0.612	0.921	0.754	0.327	0.439

## Data Availability

The dataset used in this study is publicly available online at https://www.cse.wustl.edu/~jain/ehms/index.html (accessed on 10 August 2026).
